# A small-molecule chemical interface for molecular programs

**DOI:** 10.1093/nar/gkab470

**Published:** 2021-07-05

**Authors:** Vasily A Shenshin, Camille Lescanne, Guillaume Gines, Yannick Rondelez

**Affiliations:** Laboratoire Gulliver, CNRS, ESPCI Paris, PSL Research University, 10 rue Vauquelin 75005 Paris, France; Laboratoire Gulliver, CNRS, ESPCI Paris, PSL Research University, 10 rue Vauquelin 75005 Paris, France; Laboratoire Gulliver, CNRS, ESPCI Paris, PSL Research University, 10 rue Vauquelin 75005 Paris, France; Laboratoire Gulliver, CNRS, ESPCI Paris, PSL Research University, 10 rue Vauquelin 75005 Paris, France

## Abstract

*In vitro* molecular circuits, based on DNA-programmable chemistries, can perform an increasing range of high-level functions, such as molecular level computation, image or chemical pattern recognition and pattern generation. Most reported demonstrations, however, can only accept nucleic acids as input signals. Real-world applications of these programmable chemistries critically depend on strategies to interface them with a variety of non-DNA inputs, in particular small biologically relevant chemicals. We introduce here a general strategy to interface DNA-based circuits with non-DNA signals, based on input-translating modules. These translating modules contain a DNA response part and an allosteric protein sensing part, and use a simple design that renders them fully tunable and modular. They can be repurposed to either transmit or invert the response associated with the presence of a given input. By combining these translating-modules with robust and leak-free amplification motifs, we build sensing circuits that provide a fluorescent quantitative time-response to the concentration of their small-molecule input, with good specificity and sensitivity. The programmability of the DNA layer can be leveraged to perform DNA based signal processing operations, which we demonstrate here with logical inversion, signal modulation and a classification task on two inputs. The DNA circuits are also compatible with standard biochemical conditions, and we show the one-pot detection of an enzyme through its native metabolic activity. We anticipate that this sensitive small-molecule-to-DNA conversion strategy will play a critical role in the future applications of molecular-level circuitry.

## INTRODUCTION

The field of molecular programming (MP) develops systematic methods for the synthesis of complex molecular systems with desired behaviours or functions, *in vitro* (1). In particular, the use of synthetic DNA oligonucleotides as information-encoding molecules has allowed fast progress in our ability to construct multi-species reaction networks. These systems are able to emulate, *in vitro*, electronic circuits (2,3), cellular regulation networks (4,5), ecological webs (6,7) or neural architectures (8). The modularity and chemical simplicity of this approach allow rational and scalable circuit assembly. Analog molecular computation, for example, was demonstrated by Song *et al.* in the case of polynomial functions (9).

One critical limit of most *in vitro* DNA-based approaches, however, is that they only accept inputs presented as nucleic acid species. In contrast, natural networks, as well as many *in vivo* synthetic circuits, accept a variety of chemicals, including small molecules, as inputs. For example, the historically important *lac* operon is a small regulatory circuit that optimises the metabolic response of a bacteria on a 2-dimensional glucose/lactose concentration space (10). Synthetic biology also builds on the rich catalogue of regulatory elements, such as allosteric regulators or riboswitches, to engineer a variety of circuits responding to small-molecule inputs (11,12).

Following the biological inspiration, early studies explored two strategies, a *direct* or an *indirect* one, to connect nucleic acid reactions networks and small molecule chemistry. The direct strategy leverages specific, sequence-dependent DNA or RNA folds, termed aptamers (13,14), that display tailored binding affinity for a given target molecule (15). Inserted in the synthetic strand design, these elements can modulate the circuit's reactivity according to their ligand's concentration (16). The indirect approach uses an intermediate molecule to bridge nucleic and small molecule chemistries. A prominent example is provided by some natural or engineered (17) transcription factors (TFs), which recognise and bind specific sequences in double-stranded DNA, and whose binding affinity is strongly modulated by small effectors. Both approaches have allowed the building of simple *in vitro* sensors (18–20). For example, L-tryptophan has been detected via the release of TF-induced inhibition of the rolling circle amplification (RCA) on a template displaying the operator site (18). Using an aptamer Oishi *et al.* designed an ATP-sensitive circuit embedded in a DNA gel (21).

However, little is known concerning the potential of TFs to provide a general interface between small molecule stimuli and DNA-based molecular circuits.

In this work, we set out to develop a versatile approach that both leverages state-of-the-art capabilities in terms of MP, in particular neural architectures (8), and connects it to physiologically relevant concentrations of small molecule inputs (i.e. micro to millimolar). We show the benefit of the molecular signal-processing layer, which we use to invert the signal, or generate multi-input classification. In addition, we adapted the system to work in mild and bio-compatible conditions, thereby increasing the range of possible applications of these circuits. We demonstrate this last advantage by building a DNA circuit that senses the activity of enzymes, whose catalytic action is in principle completely orthogonal to the chemistry of nucleic acids.

## MATERIALS AND METHODS

### Oligonucleotides

High-performance liquid chromatography-purified DNA oligonucleotides were obtained from biomers.net. The sequences are listed in Supplementary Table S2. Most templates were modified at their 5′-end by phosphorothioate bonds and at their 3′-end by a phosphate group to respectively prevent their degradation by the exonuclease and their extension by the polymerase. An exception are the protein sensing templates, which were not susceptible to the issue. Reporter template (rT) was conjugated to Atto663 and BHQ2 at 5′- and 3′-ends (instead of the phosphate group), for fluorescent reporting. Thermodynamic parameters were calculated using the DINAMelt server.

### Protein purification

LacI and TrpR CDSs were cloned from NEB 5-alpha competent cells via Gibson assembly and expressed in NEB T7 express on pIVEX-based vector containing C-terminal His-tag kindly provided by Rémi Sieskind. Purification was carried out at 4°C on GE Healthcare Life Sciences His GraviTrap TALON columns and stored in 20 mM Tris–HCl pH 8.0, 0.5 M NaCl, 50% glycerol prior to use. TrpB variants were purified in a similar manner but using KRX cells. The *Pf*B gene was ordered as a gBlock and NEB KLD kit was used to generate point mutations in genes from both organisms (See Supplementary Note 6 for cloning materials). Enzymatic activity of *Ec*B L-trp synthesis was confirmed through HPLC evaluation. Briefly, 200 mM phosphate buffer pH 8.0 with 3 mM l-serine, 2.4 mM indole, 40 μM pyridoxal 5'-phosphate (PLP) was prepared. 5.6 μM *Ec*B was added for an overnight incubation at 37°C along with negative control that contained no enzyme and was not incubated. Enzyme was filtered out using Amicon Ultra 0.5 ml centrifugal filters MWCO 3kDa. Samples were run on 2.1 × 100 mm C-18 silica column with acetonitrile/water: 0% acetonitrile for 1 min, 0% to 100% over 5 min, 100% for 4 min and then a wash with water for 10 min before the following sample. Using absorbance measurements at 290 nm, the negative control only contained a peak at 5.36 min, corresponding to the indole standard, whilst overnight incubation produced a single peak at 3.24 min, corresponding to L-trp standard.

### Reaction assembly

Reactions were performed in a buffer containing 20 mM Tris–HCl pH8.9, 10 mM (NH_4_)_2_SO_4_, 40 mM KCl, 10 mM MgSO_4_, 10 mM NaCl, 2 μM Netropsin (Sigma-Aldrich), 200 μg ml^–1^ BSA (New England Biolabs, NEB), 0.1% Synperonic F108 (Sigma-Aldrich), 0.4× EvaGreen dye (Biotium) and 25 μM of each dNTP. All experiments also contained Bst DNA polymerase, large fragment, the nicking endonucleases Nb.BsmI and Nt.BstNBI (all from NEB) respectively used at 20, 200 and 0.5 U ml^–1^ (0.25, 2 and 0.005% final dilutions of the commercial stock solutions). The thermophilic 5′ → 3′ exonuclease ttRecJ was purified in the laboratory, stored in Diluent A (NEB)+ 0.1% Triton X-100 at a concentration of 1.53 μM and used at 23 nM. Where relevant, LacI was added at 110 nM and TrpR at 210 nM of dimer.

Reactions were assembled in a total volume of 10 μl and run at 37°C in a CFX96 real-time PCR detection system (Biorad). As reported for polymerase/nickase amplification schemes (27,72), we sometimes observed the emergence of template-independent amplification products at longer incubation times. These parasitic reactions—that may occur from both the high or low state of the switches—are easily distinguished from their fluorescent signature and were not taken into account for the analysis.

**Figure 1.**
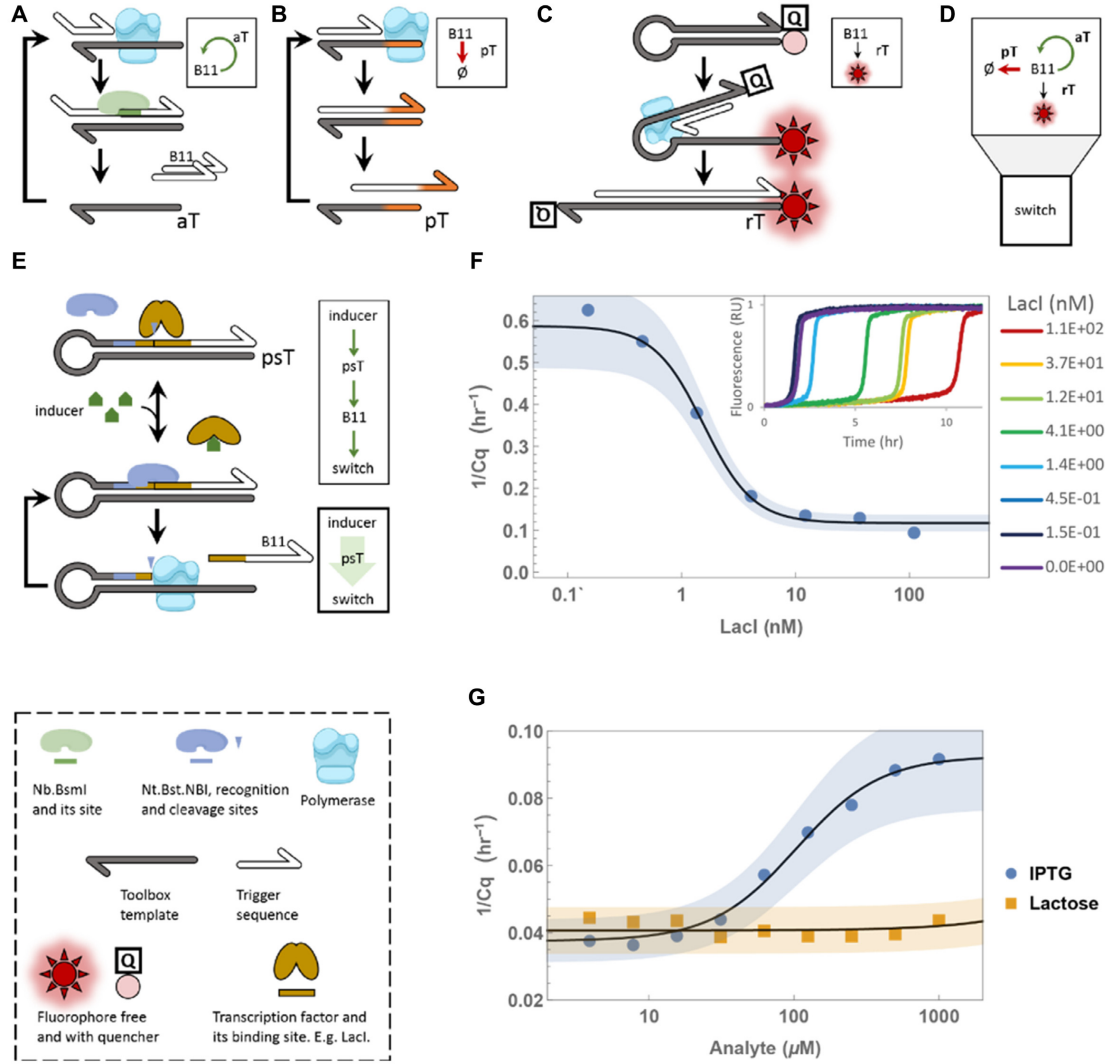
PEN-DNA toolbox elements for small molecule sensing. Polymerase, exonuclease and nickases (PEN) are used to drive a series of modules. (**A**) The autocatalytic module where ssDNA signal B11 binds the dual-repeat template aT, is elongated and nicked, thus releasing two identical triggers. (**B**) The pseudo-template module where the signal binds pT, is extended with a TT 3′-end (orange), and released. The TT-tailed signal cannot initiate an aT cycle and will eventually be degraded. The pT reaction is faster than the aT one, but has limited capacity, leading to an adjustable threshold effect (**C**) Reporting module where the signal is extended over the hairpin structure, therefore dequenching a fluorophore, giving rise to fluorescence. (**D**) The combination of aT, pT and rT provides a bistable amplification switch, whose state is controlled by the concentration of B11 signal. (**E**) TF (e.g. LacI) occludes the Nt.BstNBI nicking site on psT blocking its ability to produce signal via sequential nicking and polymerisation cycles. Inducer addition results in TF unbinding and signal production. (**F**) Time-response to a range of LacI dimer added to the switch with psT_LacI_. Inset – normalised fluorescent time traces. (**G**) Time-response to a range of IPTG (circles) and Lactose (squares) added to the switch with psT_LacI_ and LacI mixture.

**Figure 2.**
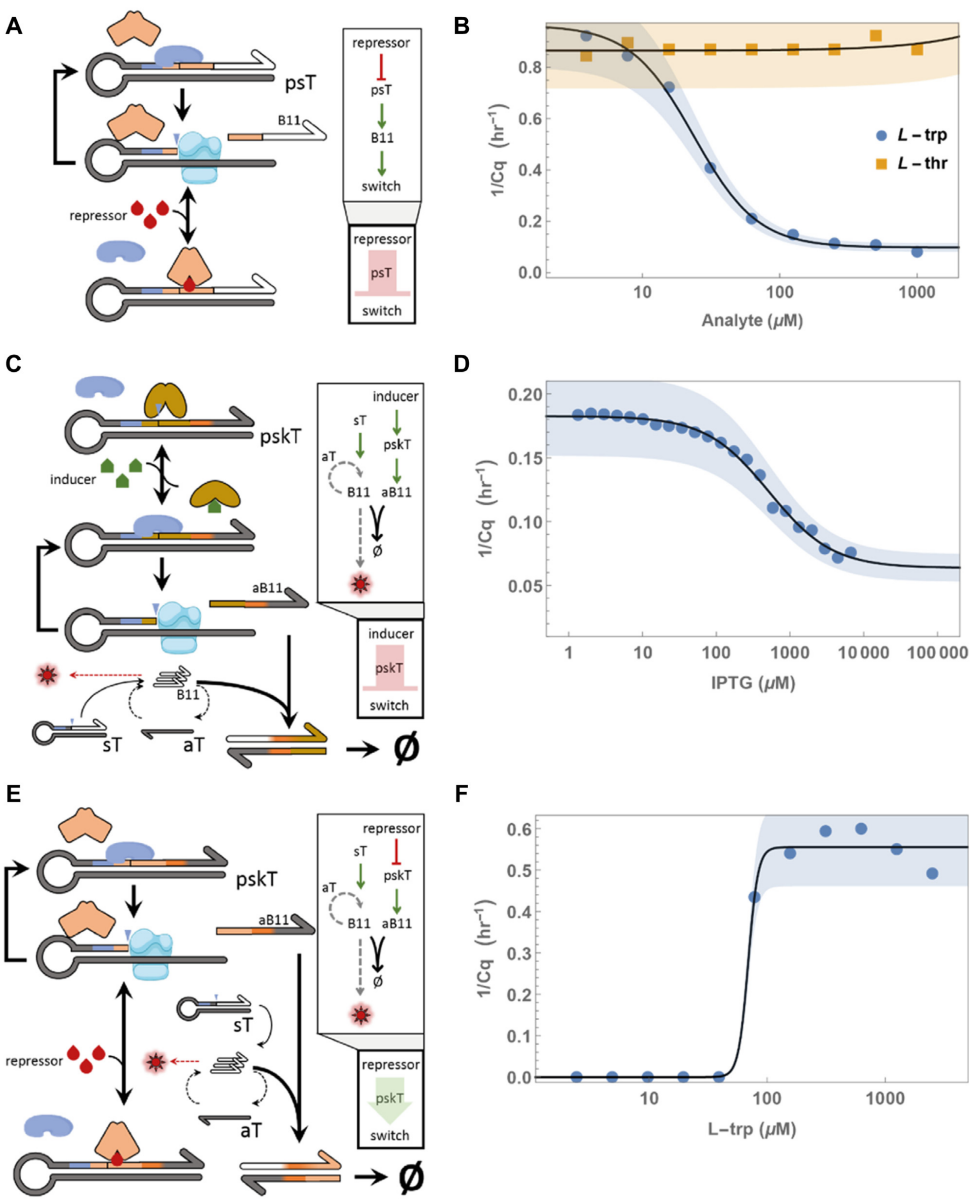
PEN-DNA small molecule sensing modules and their dynamics. (**A**) TF blocks the Nt.BstNBI site on psT upon repressor addition and stops B11 production via sequential nicking and polymerisation cycles. (**B**) Time-response to a range of L-trp (circles) and L-thr (squares) added to the switch with TrpR and psT_TrpR_ mixture. (**C)** TF occludes the Nt.BstNBI nicking site on pskT, blocking its ability to produce antiB11 (aB11) via sequential nicking and polymerisation cycles. Inducer addition results in TF unbinding and aB11 production leading to B11 consumption and switch deactivation. (**D)** Time–response to a range of IPTG added to the switch with pskT_LacI_ and LacI mixture. (**E**) aB11 is produced from pskT via sequential nicking and polymerisation cycles and the switch is initially deactivated. Repressor addition results in TF binding and aB11 production disruption leading to switch activation. (**F**) Time-response to a range of l-trp added to the switch with pskT_TrpR_ and TrpR mixture.

**Figure 3.**
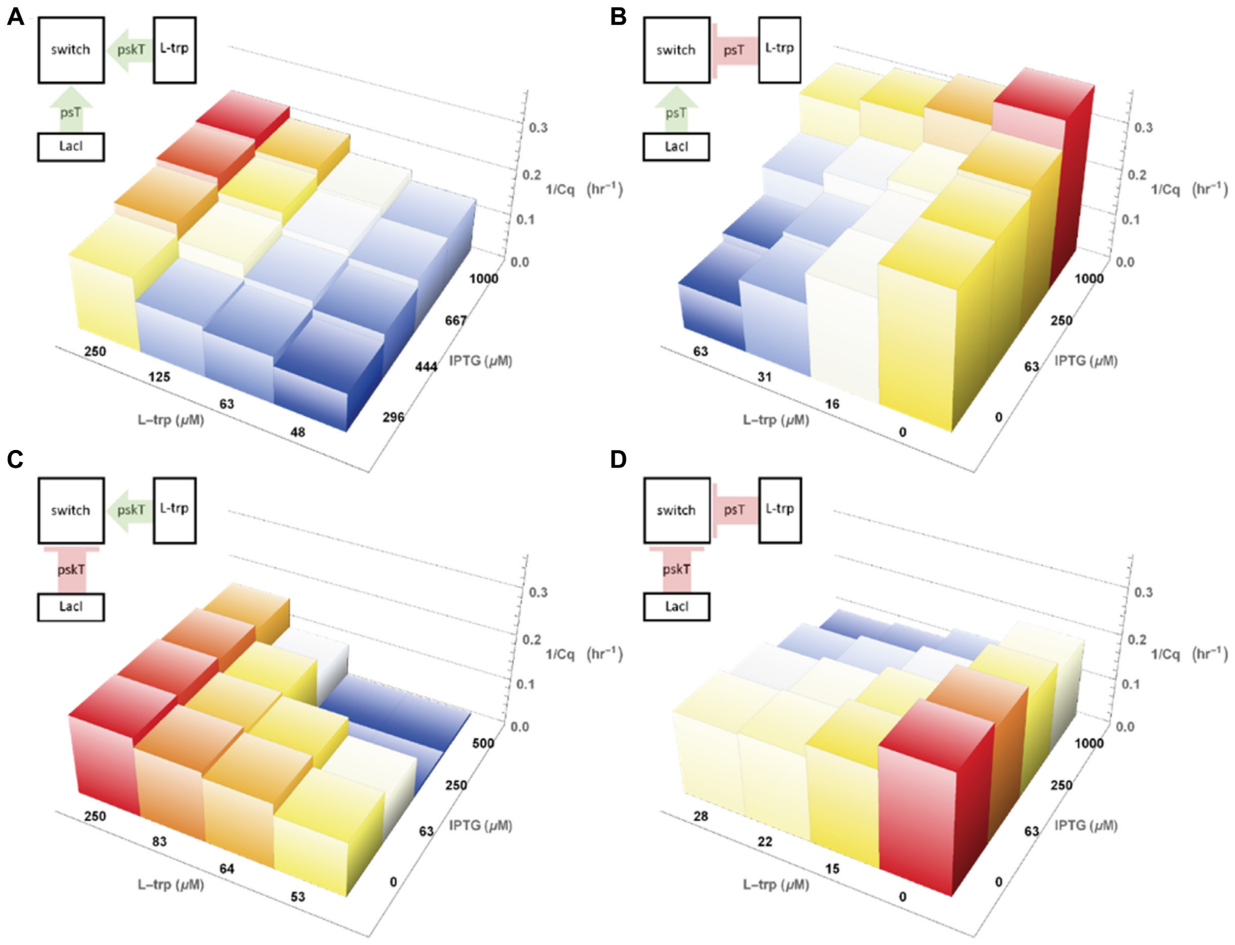
Combinations of protein sensing (killer) templates with ranges of concentrations of two inputs and LacI with TrpR in all samples. Circuit schematics are shown above the figures. Colour range represents relative 1/Cq variation within individual plots. (**A**) pskT_TrpR_ and psT_LacI_ for l-trp inverted sensing, IPTG direct sensing. (**B**) psT_TrpR_ and psT_LacI_ for l-trp direct sensing, IPTG direct sensing. (**C**) pskT_TrpR_ and pskT_LacI_ for l-trp direct sensing, IPTG inverted sensing. (**D**) psT_TrpR_ and pskT_LacI_ for l-trp inverted sensing, IPTG inverted sensing.

**Figure 4.**
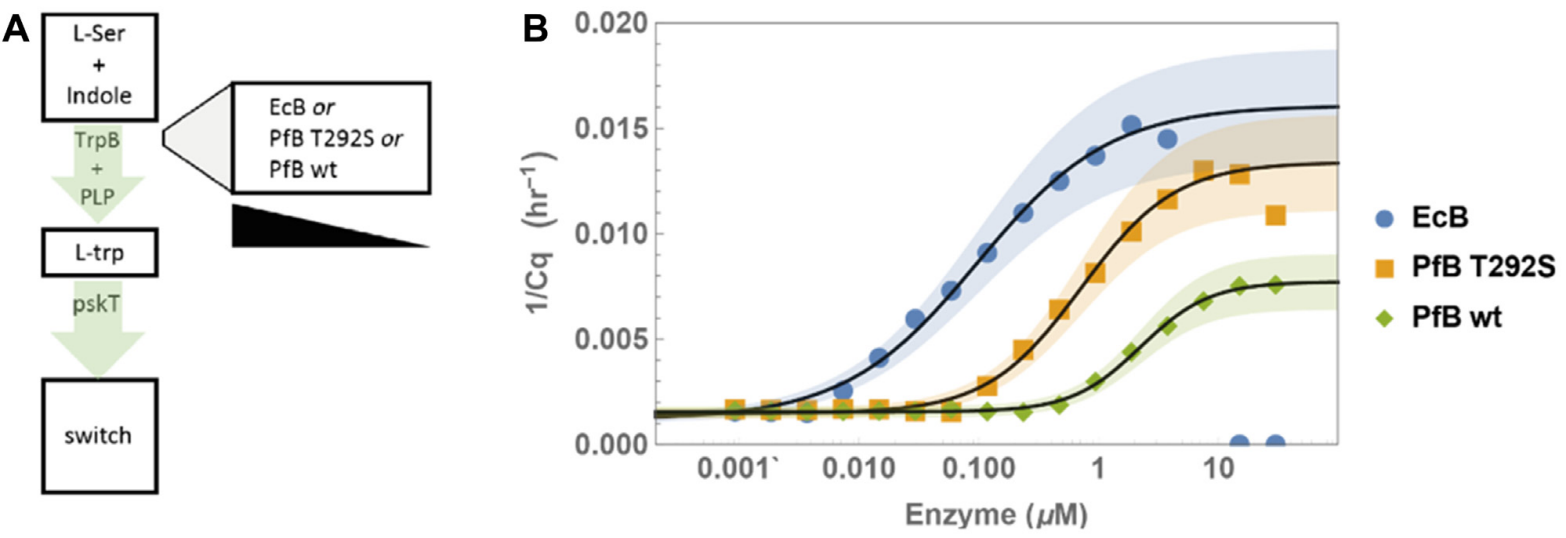
Sensing of L-trp production *in situ*. (**A**) L-serine and indole are converted to L-trp by one of the TrpB variants listed in the box (from *E. coli* or *P. furiosus*), a metabolic activity that is sensed *in situ* by the switch. (**B**) Switch response to L-trp production by various concentrations of EcB (circles), PfB T292S (squares), PfB wt (diamonds).

Concentrations of aT and rT were fixed at 50 nM and 10 nM accordingly as in previous works (22–25), sT 8 pM (minimally saturating concentration) for pskT-only circuits, pT at 7 nM, unless otherwise specified whilst for the remainder:

Figure 1: F. psT_LacI_ 5 nM; G. psT_LacI_ 2.5 nM.
Figure 2: B. psT_TrpR_ 1 nM; D. pT 4 nM, pskT_LacI_ 10nM; F. pT 4 nM, pskT_TrpR_ 10 nM.
Figure 3: A. pskT_TrpR_ 10 nM, psT_LacI_ 10 nM; B. psT_TrpR_ 0.13 nM, psT_LacI_ 2.5 nM; C. pT 4 nM, pskT_TrpR_ 1.5 nM, pskT_LacI_ 7.5 nM; D. pT 1 nM, psT_TrpR_ 0.063 nM, pskT_LacI_ 40 nM.
Figure 4: 50 nM of rT was used, as well as 1.5 times concentration of Nb.BsmI and 1.8 times concentration of Bst DNA polymerase, 50 nM of TrpR dimer. pT 5nM, sT 2.5 nM, 10 mM of L-serine, 80 μM PLP, 5 mM indole, pskT_TrpR_ 10 nM.

### Data acquisition and analysis

A template conjugated to Atto663 fluorophore on the 5′-end and BHQ2 quencher on 3′-end (rT) was used to specifically monitor switch activation. The signal of the fluorophore is quenched when the template is in a hairpin conformation, which opens upon trigger binding and extension. Nonspecific reactions were monitored from the fluorescence signal produced by a double-stranded DNA intercalating dye (EvaGreen). Fluorescence signals were acquired every minute in the channels corresponding to the two dyes present in solution. ‘Start time’ was derived from Atto663 time traces through a workflow implemented in Excel. In brief, each trace is normalised so that fluorescence values lie between 1 and 0; differences between 2 points 10 min apart are then calculated (last 10 min are excluded) and the lower time-point corresponding to maximum difference value is extracted. The data are manually curated to identify flat traces with no distinctive switch-driven fluorescence increase which are assigned 1/Cq = 0. Where appropriate, Hill Function fitting was performed using GraphPadPrism7. The estimated error determination is based on the maximum single point error for the extraction of Cq values in triplicate experiments performed for Figure 1G, and applied to all other plots.

## RESULTS AND DISCUSSION

### Design of the bistable amplification switch

We started with the PEN-DNA framework (22), a powerful molecular programming language which has allowed the rational building of a variety of molecular systems, including batch oscillators (23), programmable pattern formation (24), or ultrasensitive multi-input sensors (25). The PEN-DNA employs Polymerase, Exonuclease and Nickase (PEN) enzymes as the chemical machinery. Short protected single-stranded oligos (templates) with an input-output structure provide instructions. Data flows are implemented via the endogenous production, exchange and decay of smaller strands of DNA called signals. The sequence of these signal strands is designed in such a way that (i) the *T*_m_ of a single hybridised signal is similar to the reaction temperature, yielding dynamic binding and unbinding to templates and (ii) it carries recognition sequence site enabling productive nicking/polymerisation cycles on the template.

Here, we build on three essential modules from the PEN-DNA toolbox: the first one is the autocatalytic module (aT, for autocatalytic template) (Figure 1A), whose encoding oligo consists of a dual repeat of the complement of an arbitrary ‘signal’ sequence. When a cognate trigger binds to an aT, it is extended by the polymerase and the resulting full duplex is nicked, releasing two copies of the signal. aTs thus provide positive feedback loops and exponential amplification of signals. However, as noted in a number of reports (26,27), this motif is intrinsically sensitive to spurious initiation.

The second module, a pseudotemplate (pT), provides a solution to this instability issue. pT (Figure 1B) also has a trigger binding site, which is followed by a short poly-T tail (in this paper 2 bases). Additionally, the binding energy of the trigger for pT versus aT is biased in favor of pT by removing a few bases to the 3′ extremity of the aT. At low concentration, triggers thus bind preferentially to pT, are extended by a short tail, which deactivates them. Above a certain concentration threshold, this sink gets saturated and exponential amplification ensues. The combination aT/pT thus creates a bistable switch that can be adapted to sensitive detection of a variety of inputs (25,28,29).

The dynamics of these systems can be observed with non-specific dsDNA fluorescent dyes or by a variety of sequence-specific fluorescent reporters based on DNA-dye conjugates. Here we will use a trigger-specific fluorescent probe, we called reporter template (rT) (Figure 1C). It consists of a hairpin-structure DNA with the two ends carrying a quencher and a fluorescent dye. Upon binding and extension of the trigger, the hairpin structure is disrupted, increasing the separation between the two moieties and giving rise to fluorescence. Since the trigger is short, this form is relatively unstable, but it is captured by the polymerase, which extends the trigger along the probe, thus making the unquenching reaction essentially irreversible. We shall refer to this 3-module assembly (aT, pT, rT) as a ‘switch’ throughout the text (Figure 1D).

Similar switches have already been used for the sensitive detection of nucleic acids, down to the femtomolar range (25,28). However, these circuits were typically run at temperature ranging from 45 to 50°C, which is not appropriate if one wishes to use them to detect, or in combination with, protein components coming from a mesophilic organism. The first step was therefore to adapt the working temperature of the switches down to 37°C. Although the PEN enzymes are thermophilic, with optimal temperature ranging from 55°C to 70°C, we found that shortening the signal sequence and decreasing its melting temperature to around 26°C could still provide reasonable kinetics. We then optimised the concentration of switch components to achieve robust bistability, yet reasonable threshold of activation (see Supplementary Note 1, Tables S1 and S2 for details).

### Design of a sensing module for sensitive small biomolecule detection

As a first step to connect the PEN-based networks to non-DNA inputs, we designed a new module called the protein sensing template (psT) (Figure 1E). A psT is a hairpin-shaped structure containing in the stem a nickase site followed by two variable elements: a protein binding site (operator) and an output-encoding sequence. For the nickase Nt.BstNBI, the cleavage site is located 4 bp downstream of the recognition site, so it can be designed to overlap with the operator site without sequence constraints (Supplementary Figure S3). In absence of the cognate operator-binding protein, this template acts simply as a constant source of its output sequence, through self-primed extension-and-nicking cycles. Here, the output sequence is the switch's ‘signal’ sequence, so a sufficient concentration of free psT will eventually turn the switch on. When the DNA-binding protein attaches to the psT, its blocks access for the nickase because of steric hindrance. In that configuration, the output production rate is expected to drop, and the switch start-up time to be delayed or abolished (see Supplementary Figure S4 for full circuit design).

To validate this strategy we expressed and purified *Escherichia coli* LacI, a well-characterised transcription factor (30). LacI dimers bind DNA containing one of the three naturally occurring operator sequences with an affinity ranging from 1 pM to 100 nM (31–34). In parallel, we designed the corresponding psT, with the particular output ‘signal’ sequence chosen—‘B11’ (an 11 nucleotide sequence) and LacI O_1_ sequence—the operator sequence with the lowest *K*_d_.

We combined psT_LacI_ with the switch and tested a range of LacI concentrations. As expected, we observed a direct effect on the switch response, with more TF leading to a delayed initiation (Figure 1F). The repressive effect seems to saturate close to 4 nM of LacI dimer, consistent with the concentration of psT (5 nM), and the reported tight affinity of the TF for its operator (35) (∼1 pM).

We define the time of switching (Cq) as the time point where the gradient of rT fluorescent trace is steepest. Since in some conditions (strongly repressed psT) the switch does not start at all in the experimental timescale, we find it useful to use 1/Cq values. This allows to represent non-starters as 0 and to assign the highest values to the fastest reactions (see Supplementary Figure S5 for details on data processing).

The affinity of many DNA binding proteins to their target site is allosterically modulated by small ligands. The corresponding psT thus also provides an opportunity to interface the circuits to these effectors. For example, the classic inducer isopropyl β-d-1-thiogalactopyranoside (IPTG) can decrease LacI affinity to DNA by over 4 orders of magnitude (35). We therefore tested whether this strategy could be used to connect PEN-DNA reactions to small molecule inputs. A range of IPTG going from 0 to 1000 μM was added to the DNA circuit described above. We observed indeed that the DNA system responds, within a couple of hours, to the various IPTG concentrations (Figure 1G). The behavior is reminiscent of the typical sigmoidal bacterial induction curve for LacI: more IPTG led to smaller Cq, that is, higher switch activity. The apparent *K*_d_, close to 100 μM, appears reasonable, provided the reported one is around 23 μM (35) at pH 9.2, close to our pH 8.9. IPTG is an analogue of naturally occurring allolactose, the true effector of this system, and we achieved similar results with the latter (Supplementary Figure S6). As a control, we used the same concentration range of lactose, also a close chemical analogue – a metabolic precursor of allolactose with no significant allosteric effect on LacI. As expected, the switch circuit did not respond at all to lactose (Figure 1G).

The results above thus suggest that the specificity of the natural allosteric transducer (36) has been maintained upon embedding into an artificial *in vitro* circuit. Using appropriate calibration curves, this isothermal DNA circuit can thus be used to specifically quantify the concentration of a DNA-binding protein, or its small molecule effector.

### Expanding the range of signal inputs

The binding of allosteric transcription factor is either activated or repressed by their ligand. While IPTG, or allolactose disrupts the LacI-DNA complex, other TFs may follow the opposite logic: for example, TrpR, the transcription factor responsible for tryptophan (trp) metabolic regulation, can only bind its operator sequence *in presence* of L-trp (37,38). To test whether this class of sensing proteins could be used with DNA-based circuits, we purified *E. coli* TrpR and designed the corresponding psT presenting the TrpR operator site and the same B11 signal as output sequence (Figure 2A). When the reaction was assembled as before, but with TrpR and psT_trpR_ as input module, we observed that the switch indeed responded to the presence of L-trp. However, the response was inverted compared to the previous case, with more L-trp leading to larger Cq. Here as well, we could confirm the specificity (39) of the sensing circuit using another amino acid as analogue: a concentration of l-threonine (l-thr) as high as 1 mM had no detectable effect on the switch. The Hill curve fitting on the l-trp range yielded a *K*_d_ value of 24 μM, again consistent with the reported value of 20 μM (40,41) (Figure 2B).

In practical applications, being dependent on the TF internal allosteric logic, i.e. its positive or negative response to the molecular stimulus, may seem inconvenient. Fortunately, the PEN toolbox offers straightforward mechanisms for logical inversion (42,43). Here we decided to replace the B11 output of the psT with the complementary sequence antiB11 (aB11). For clarity we named these repressing input modules protein sensing *killer* Template (pskT), in line with recently described killer Templates (kT) (25) (Figure 2C, E). At the same time, the mixture was complemented with an unconditional mechanism to produce the signal B11, which we call a source template (sT). An sT is similar to a psT but lacks the operator element. Triggering of the switch is then controlled by the opposing effects of sT and pskT, whose balance is shifted by the TF binding on pskT. Figure 2D, F shows the result of inverted LacI and TrpR-based sensors using this approach: the cognate small molecule now acts as inhibitor (respectively activator) of the DNA-based bistable switch. See Supplementary Note 2 and Supplementary Figures S7 and S8 for details about sT, psT and pskT function and design.

In these systems, while signal inversion behaved as expected, the apparent *K*_d_ values increased: *K*_d_ were around 500 and 69 μM, for LacI and TrpR-based systems respectively. We attribute this decrease in performance to the fact that pskT product has to overcome the continuous production of triggers by the sT, in order to maintain the switch in an OFF state. Interestingly however, the competitive mechanism implemented by this additional inversion layer could result in a much sharper transition than the corresponding direct design. In the case of TrpR, we observed a Hill coefficient shifting from 1.9 to 10.5. This observation is reminiscent of similar ultrasensitive behavior in system based on inhibitor titration (44,45), and shows that the DNA circuit can also be used to reshape the functional response of the allosteric element (see also Supplementary Note 3).

### Circuit integration

Transducing small molecule concentration into DNA reactions in principle allows us to deploy the full toolbox of molecular information processing, including logic functions, pattern recognition, signal amplification, etc, to the sensing of metabolites. To demonstrate this idea, we created two-input molecular classifiers, based on IPTG and l-trp. Using the logical inversion mechanism reported above, we designed the four possible 2D systems (Figure 3).

Combining two input channels into an integrated response, whatever the logic, implies that the time scales and reaction strength are adequately matched. Fortunately, in DNA-based chemical reaction networks these parameters are controlled by the reaction kinetics, and ultimately by the encoding DNA concentrations. We could therefore balance the systems, by adjusting psT/pskT concentrations to change the inputs strength, and pT to adapt switch sensitivity. Given these adjustments, we obtained a series of plots showing rotational symmetry. This tunability is both useful for rapid rewiring of a circuit and for adaptation to different transcription factors. In the plots shown in Figure 3, we tuned the system to obtain an approximate weighted log-sum on the inputs, where the weight can be positive or negative, but other responses are possible. The weight and shape of the contribution of a particular input can be varied, which can be used to prioritise one analyte over another or binarise the response (see Supplementary Note 4, for examples of different 2D sensing functions). This again demonstrates the benefit of the DNA circuit as a versatile signal-processing layer acting to modulate the intrinsic molecular characteristics of the functional components – here, the TFs.

### In situ metabolic sensing

One step upstream of the small molecule world stand the metabolic enzymes that create, consume or modify them. For example, l-trp is the product of Tryptophan synthase subunit B (TrpB), which synthesises it from indole and l-serine. This reaction is usually coupled with indole production in subunit A of the TrpAB dimer, and is slow in the absence of TrpA (46). Standalone TrpB, which could be used in a variety of drug synthesis schemes, has therefore recently been the focus of rich directed evolution studies (47–50) and provided a suitable target. Some of the improved variants reported are *Escherichia coli* TrpB^M149T N171D^ (*Ec*B from now on) and *Pyrococcus furiosus* TrpB ^T292S^ (from now on *Pf*B T292S, or *Pf*B wt for wildtype). We thus purified these variants and endeavoured to optimise our reaction assembly to accommodate for enzymatic synthesis as well as L-trp product sensing, over a range of enzyme concentrations (Figure 4A).

As we could expect, for equivalent enzyme concentrations and at 37°C, we found that the mesophilic homologue performed best, followed by enhanced *P. furiosus* variant (Figure 4B). TrpB co-factor pyridoxal 5'-phosphate (PLP), as well as the reactants, do not seem to affect the circuit. Only very high enzyme concentrations (>10 μM) showed a non-specific disruptive effect to the DNA circuit.

## CONCLUSION

This work demonstrates that DNA-based molecular programs can be interfaced with small molecule inputs using engineered modular converter elements. Compared to other small molecule sensing approaches (e.g. optical (51) or electrochemical (52)), which provide physical readouts, our approach uses chemical-level transducers. Once the molecular signal is forwarded to the DNA layer, a wide range of off-the-shelf molecular programming techniques and modules can be used for signal processing *in moleculo*. Here, we used signal amplification, thresholding, shaping, inversion and integration. This approach is modular and tunable, as DNA reactions can be designed with limited cross-over, between themselves or with other chemistries. It is thus possible to add a branch – or replace one – with the operator sequence and the TF of interest, without the need to redesign the other components.

A limit of this design, however, may lie in its dependence on existing allosteric DNA-binding proteins. Fortunately, large libraries of natural and artificial TFs are readily available, due to their common use in a variety of applications (17,53,54). Faulon *et al.* for example demonstrated the modular use of various TFs for signal integration in bacterial context (55,56), and compiled a catalogue containing over 4600 entries of characterised TFs (57). This is complemented with a CAD tool that suggests enzymatic pathways to convert small molecules of interest to those detectable by a TF (58). The latter is also compatible with our circuit.

Transcription factors are usually very specific with respect to their effectors and operators, and could therefore be used to create highly multi-dimensional metabolome profiling networks. Concerning non-biologically relevant molecule sensing, we note that a number of studies have reported a surprising mutational plasticity of natural allosteric TF (59). TrpR for example, can be repurposed from L-trp to specific bromotryptophan sensing with only a few mutations (60). Although we have focused here on small organic molecules, a number of transcription factors are sensitive to metal ions (61), pH (62) or physical signal (63), widening the range of potential inputs. Likewise, as we show, TFs themselves can be targeted for detection (Figure 1F and Supplementary Note 3). In principle, the modularity of the design makes it compatible with design automation, which could leverage the existing databases and computational tools (53,54). Finally, the protein mediated strategy explored here is not limited to transcription factors. Split DNA- or RNA-processing enzymes, for example RNA polymerase (64) or CRISPR–Cas9 (65), can be made sensitive to a variety of ligands, and mediate their presence to a DNA/RNA circuit. Similarly, optogenetics explores strategies to modulate genetic processes using light (66), which could be reused *in vitro*.

In-emulsion molecular programs offer massive assay parallelisation at little extra cost, provided all operations are performed autonomously in the closed microcompartment (67). We therefore envisage the use of our sensors in applications involving high-throughput screening. For example, metabolic profile of a collection of cells distributed in emulsion could be examined on single-cell basis for millions of cells in parallel, and combined with ultrasensitive miRNA quantification (28). Libraries of enzymatic variants could be assayed for activity by distributing the cells containing mutant pathways or individual DNA fragments coupled to their protein products in emulsion.

Although this paper explores the chemical interface upstream of the DNA circuit, the downstream connection will prove equally important for ‘real-world’ applications (68). For example, the actuation of enzymes with DNA has been demonstrated (69) and implemented in particular as an output of PEN-DNA modules (70). Another option is the control of genetic replication processes, which open the route to programmable and autonomous directed evolution protocols (71). As a proof of principle, by replacing the L-trp circuit's fluorescent output by cognate primers production, we created a one-pot circuit that connects TrpB activity to polymerase chain reaction (PCR) gene amplification (Supplementary Note 5). Such a circuit brings the possibility of autonomous and programmable selection of metabolic enzymes.

Overall, our generic approach makes molecular programming strategies relevant to a new range of biological metabolite signals. We believe this may open innovative application areas, such as directed evolution and point-of-care medical diagnostics, through providing parallelised molecular level computation on molecular inputs.

## DATA AVAILABILITY

The data that support the findings of this study are available from the corresponding author upon request. Sequences for synthetic oligonucleotides and plasmids are provided in the Supporting information.

## Supplementary Material

gkab470_Supplemental_File
