# Supplementary material for: A small-molecule chemical interface for molecular programs
Source: Nucleic Acids Res. 2021 Jul 5;49(13):7765–74. doi: 10.1093/nar/gkab470 (PMC8287923; doi:10.1093/nar/gkab470)
Supplement: gkab470_Supplemental_File [file gkab470_supplemental_file.pdf]

A small-molecule chemical interface for molecular programs.

Vasily A. Shenshin, Camille Lescanne, Guillaume Gines, Yannick Rondelez*

Laboratoire Gulliver, CNRS, ESPCI Paris, PSL Research University, 10 rue Vauquelin 75005 Paris (France).

Supplementary Information

Table of contents

Supplementary Note 1. On switch optimization for working at 37 °C.................................... 4

Supplementary Figure 1. Switch$_{50}$ performance of a range of temperatures. 4

Supplementary Figure 2. Fluorescence time-trace of the switch with a range of pT concentrations................ 5

Oligos used in switch$_{50}$.. 5

Supplementary Table 1. Oligos used in switch$_{50}$ as previously reported. 5

Supplementary Note 2. On source template, protein sensing template and protein sensing killer template design and testing. 7

Supplementary Figure 3. In detail representation of the sensing modules used in this work. 7

Supplementary Figure 4. Extended version of Figure 1.. 8

Supplementary Figure 5. Data processing workflow for Figure 1F. 9

Supplementary Figure 6. Time response to a range of allolactose of psT$_{LacI}$-based sensor. 9

Supplementary Figure 7. Extended version of Figure 2. 10

Supplementary Figure 8. Optimizing the sT concentration 11

Supplementary Note 3. On effect of TrpR concentration on apparent K_d of pskT-based sensor. 12

Supplementary Figure 9. Effect of TrpR concentration on apparent K_d of the L-trp pskT-based sensor. 12

Supplementary Note 4. Alternative sensing function shapes to complement Figure 3. 13

Supplementary Figure 10. Alternative plots for Figure 3D with altered input balance.................................. 13

Supplementary Figure 11. Alternative shapes for the positive/negative circuit with altered input balance. . 14

Supplementary Figure 12. Alternative plots for the positive/positive circuit with altered input balance. 15

Supplementary Note 5. Enzyme activity detection and gene amplification response. 16

Supplementary Figure 13. Primer producing template (ppT) design.. 16

Supplementary Figure 14. EcB activity detection and gene amplification response...................................... 17

Novel oligos used in molecular programs of this paper. 18

Supplementary Table 2 Novel oligos used in molecular programs of this paper. 18

Supplementary Note 6. Cloning materials. 18

Table of Vectors and genes **19**

Table of primers **23**

Supplementary Note 1. On switch optimization for working at 37 °C.

Most PEN DNA based molecular networks reported so far work around 40 °C to 50 °C. Here our goal is to decrease the temperature to 37 °C, in order to bring general compatibility with most known transcription factors which come from mesophilic organisms.

Initially, we observed that simply using a reported bistable switch (1) (see **Supplementary Table 1**), originally designed to work at 50 °C (switch$_{50}$), led to loss of function when the temperature was lowered. This is shown in **Supp. Fig. 1** where the switch$_{50}$ assemblies containing (on not) the cognate source template is trialled over a range of temperatures.

The ability of the switch to start thanks to sTα appears to decay quickly as temperature decreases. At the same time, for temperature of around 42 °C and below, a non-specific start of the circuit (i.e. start in the absence of sTα) appears and becomes dominant. We concluded that at 37 °C the specific and non-specific start times would be bound to overlap.

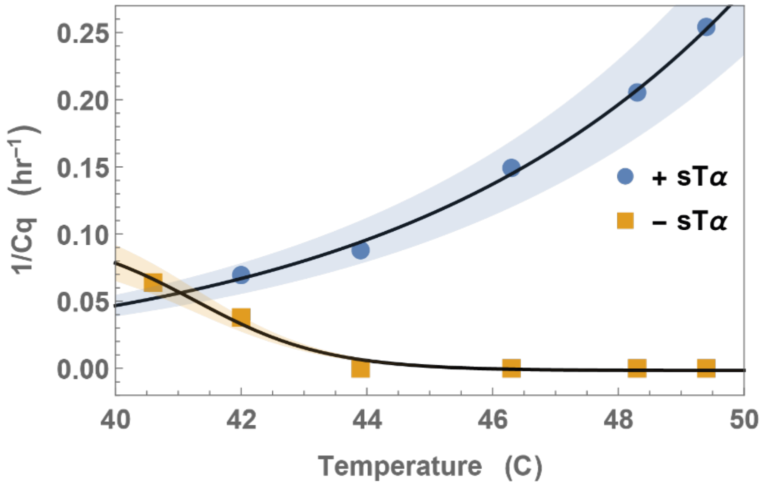

Supplementary Figure 1. Switch$_{50}$ performance of a range of temperatures. The switch was tested with (circles) or without (squares) source template at various temperatures. The expected behaviour (fast switching in presence of sT and no switching in the absence of sT) is observed down to 42 °C, although the discriminative power seems to deteriorate. Below 42°C, no discrimination was observed for Switch$_{50}$.

Method

The reaction buffer used was identical to the one reported in the main text.

However, the enzyme composition was different. Vent (exo-), the nicking endonucleases Nb.BsmI and Nt.BstNBI (all from NEB) were respectively used at 80, 300 and 100 U ml^{-1} (respectively 4%, 3% and 1% final dilutions of the commercial stock solutions). The thermophilic 5' -> 3' exonuclease ttRecJ was purified in the laboratory, stored in Diluent A (NEB)+ 0.1% Triton X-100 at a concentration of 1.53 µM and used at 22.95 nM as before. aTα was present at 50 nM, pTα at 20 nM, rTα at 50 nM and sTα at 0.5 nM (here used simply as source template).

In this experiment, Vent exo- seemed less efficient at lower temperatures. Previous PEN-DNA work has been reported with other polymerases such as Bst full length, Bst large fragment (2, 3) or Bst2.0 (warm start) (4), (all from NEB). Although, Bst Large Fragment is a thermophillic polymerase, we found, in line with others (5), that it had sufficient activity at 37 °C. Klenow was also tested in preliminary experiments, and yielded similar results. Therefore, due to its compatibility with physiological temperature, lack of 3'->5' exonuclease activity and high strand displacement activity, we selected Bst large fragment DNA polymerase for use throughout the study.

We then designed a new switch using sequences with lower melting temperature, as reported in the main text. We next had to find out the correct balance between aT and pT. Insufficient pT would result in overly weak thresholding and undesired self-starting due to spurious initiation. Excess pT would create an unnecessary drop in sensitivity as stronger stimulation would be required to start the switch.
We determined 6 nM of pT to be minimally sufficient (**Supp Fig. 2**). We chose to proceed with 7 nM of pT to increase robustness of future experiments.

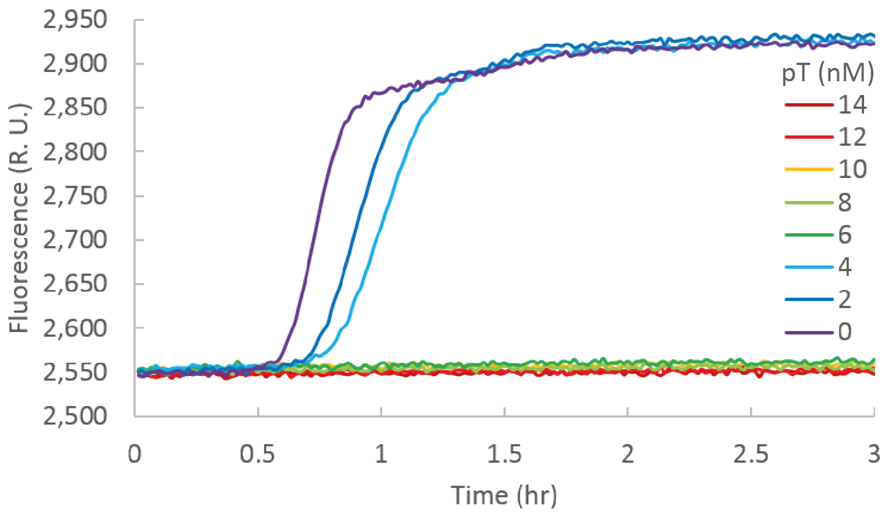

Supplementary Figure 2. Fluorescence time-trace of the switch with a range of pT concentrations. The 37 °C reaction was assembled as reported bar only containing aT and rT oligos. Samples with 0, 2 and 4 nM pT start sequentially with little delay whilst 6-14 nM pT samples remain off. The reaction was observed for 15.65 (hr) with no significant further change in fluorescence.

Oligos used in switch$_{50}$.

Name	Analogues in current work	Sequence
α	B11	CATTCTGGACTG
aTα	aT	C*A*G*T*CCAGAATG - CAGTCCAGAA
pTα	pT	T*T*T*TT-CAGTCCAGAATG
rTα	rT	Atto633*A*T*TCTGAATGCAGTCCAGAAT BHQ2
sTα	psT_{LacI}/psT_{TrpR}	TG-CAGTCCAGAA-CCTATCAATGATA-GACTC-TGCAAT TTTTATTGCAGAGTCTATCATTGATAGGTTCT

Supplementary Table 1. Oligos used in switch$_{50}$ as previously reported. Stars represent phosphorothioate modification. Fluorophore and quencher modification positions are shown for rT. Dashes are shown for ease of interpretation.

References:

1. Gines,G., Menezes,R., Nara,K., Kirstetter,A.S., Taly,V. and Rondelez,Y. (2020) Isothermal digital detection of microRNAs using background-free molecular circuit. *Sci. Adv.*, **6**, 1–9.
2. Rondelez,Y., Urtel,G., Galas,J.-C., Estevez-Torres,A., Zadorin,A.S., Gines,G., Zambrano,A. and Dilhas,V. (2017) Synthesis and materialization of a reaction–diffusion French flag pattern. *Nat. Chem.*, **9**, 990–996.
3. Montagne,K., Gines,G., Fujii,T. and Rondelez,Y. (2016) Boosting functionality of synthetic DNA circuits with tailored deactivation. *Nat. Commun.*, **7**, 1–12.
4. Gines,G., Zadorin,A.S., Galas,J.C., Fujii,T., Estevez-Torres,A. and Rondelez,Y. (2017) Microscopic agents programmed by DNA circuits. *Nat. Nanotechnol.*, **12**, 351–359.
5. Van Der Hofstadt,M., Galas,J.C. and Estevez-Torres,A. (2021) Spatiotemporal Patterning of Living Cells with Extracellular DNA Programs. *ACS Nano*, **15**, 1741–1752.

Supplementary Note 2. On source template, protein sensing template and protein sensing killer template design and testing.

We started with the design of Source Templates (sT), a hairpin structure, containing an Nt.BstNBI site followed by the B11 signal sequence. Starting from the fully elongated form, sequential nicking, B11 dissociation and polymerase extension result in constant production of B11, proportional to sT concentration. To increase circuit start efficiency, we added 2 bp at the 3'-end, to match aT but not pT. This allowed to bypass the thresholding module and increase affinity for aT. We also shortened B11 sequence on the 5'-end to compensate for 3' extension and ease dissociation from the source template.

We propagated the strategy above to Protein Sensing Templates (psTs). The same hairpin structure was taken and TrpR or LacI operator site was inserted between the Nt.BstNBI site and B11 output signal. When operator site is not occupied constant signal production occurs like above. However, this mean that the output sequence is longer as it is now catenated to a part of the operator sequence. Although this makes spontaneous dissociation unlikely (post nick), this issue is alleviated by the fact that Bst polymerase has strand displacing activity.

One can observe that the LacI operator site consists of 22 bp and is 3 bp longer than TrpR site. We think this could have contributed towards worse performance of LacI-based templates, by reducing the rate of output production of the corresponding PsT. This constraint was in part mitigated by adjusting concentrations of templates.

Protein sensing killer Templates (pskTs) were based on corresponding psT designs with replacement of output from B11 signal to antiB11 (aB11) sequence, similar to the pT sequence. As before, the resulting output contains part of the operator site as a tail.

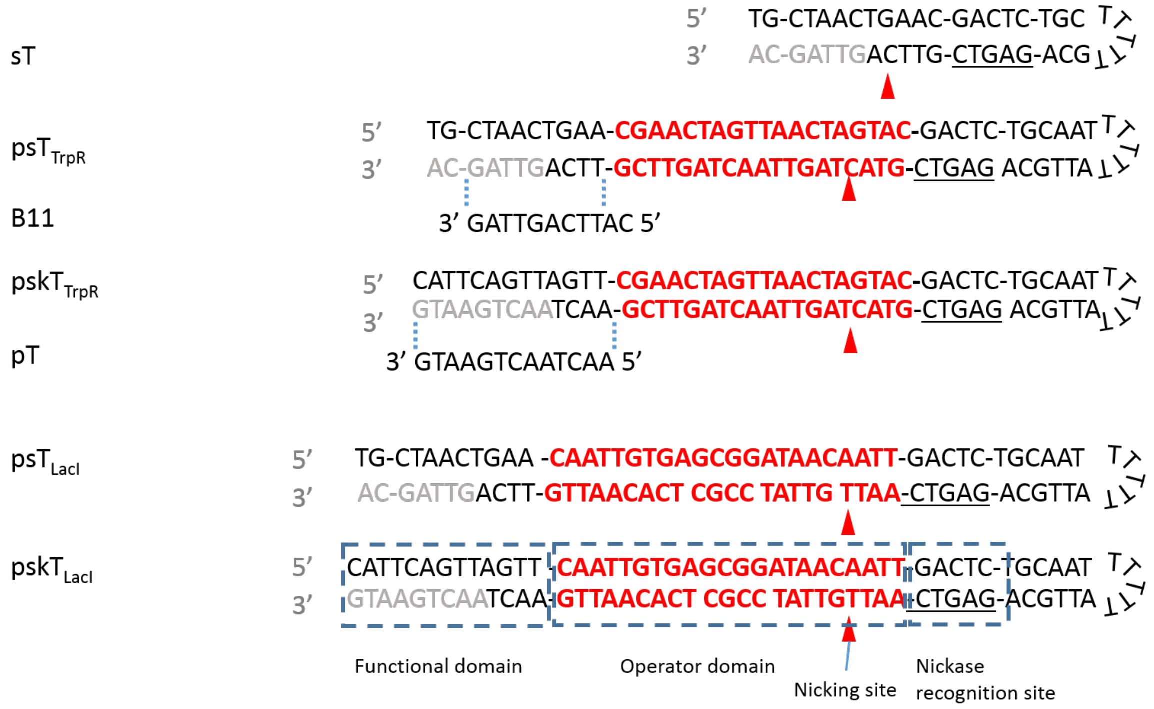

Supplementary Figure 3. In detail representation of the sensing modules used in this work. Here, going from left to right along a hairpin structure: polyT region and 6 bp, providing hairpin backbone; Nt.BstNBI recognition site (underlined); operator domain for TF binding (red), within which Nt.BstNBI nicking site is indicated (red triangle); functional region consisting of pT sequence or modified B11 signal sequence (shown standalone as well). The part of the sequence that was not chemically synthesised, but filled in by DNA polymerase upon reaction initiation, is shown in gray. Dashes are used for ease of interpretation.

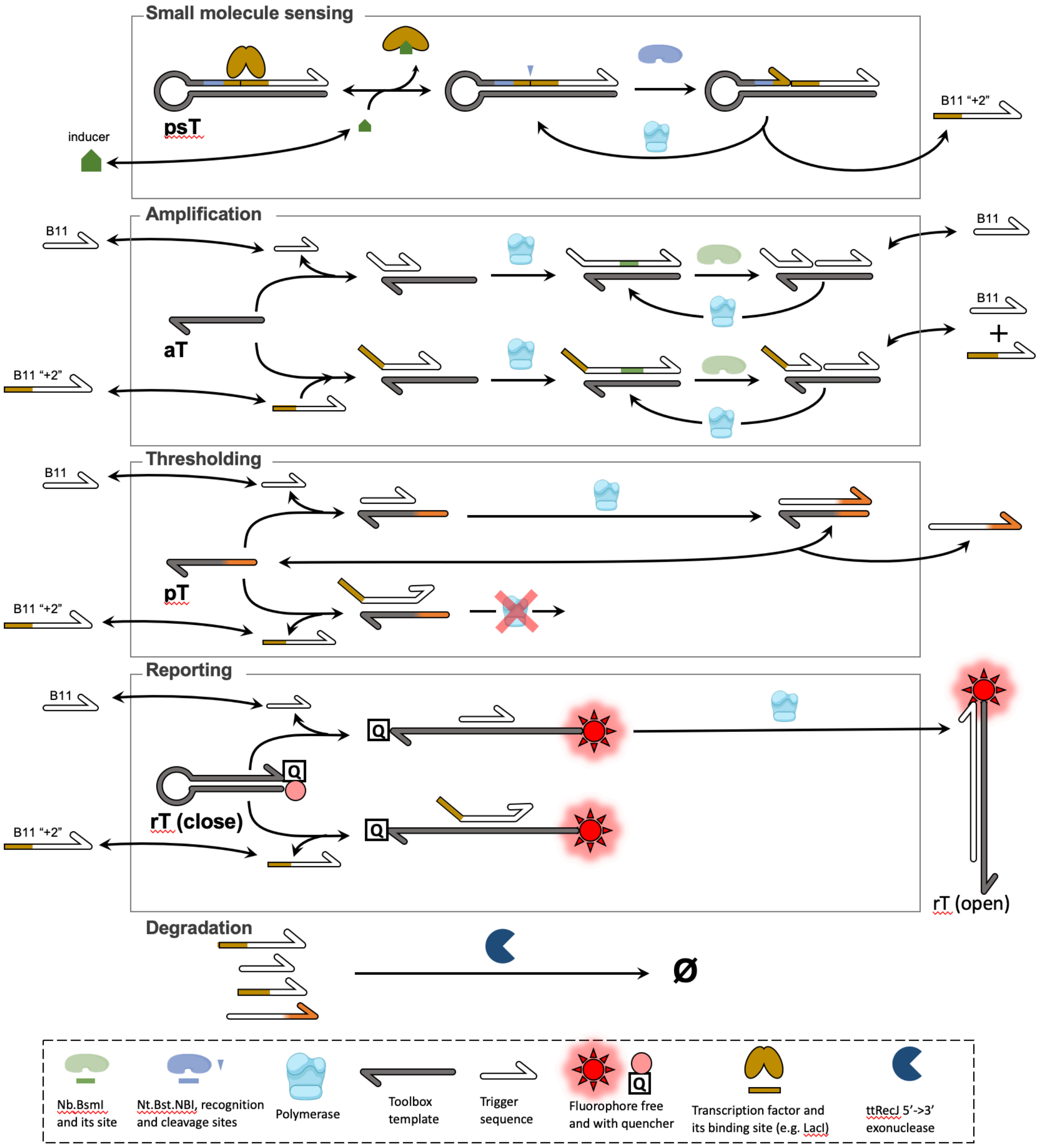

Supplementary Figure 4. Extended version of Figure 1. Small molecule sensing occurs due to unbinding of the transcription factor in response inducer addition. Sequential nicking and extension result in production of B11 "+2". B11 or B11 "+2" can bind aT and become extended. Nicking occurs. Short DNA fragments can dissociate. B11 and B11 "+2" can also bind pT. B11 "+2" is futile. B11 binds pT preferentially and is extended to contain a sequence non-complimentary to aT. B11 or B11 "+2" can also bind rT. B11 "+2" binding is futile. B11 can be extended to permanently open the rT hairpin. All "signal" strands can be degraded by ttRecJ 5' exonuclease whilst "toolbox" strands are protected due to 5' PTO modification.

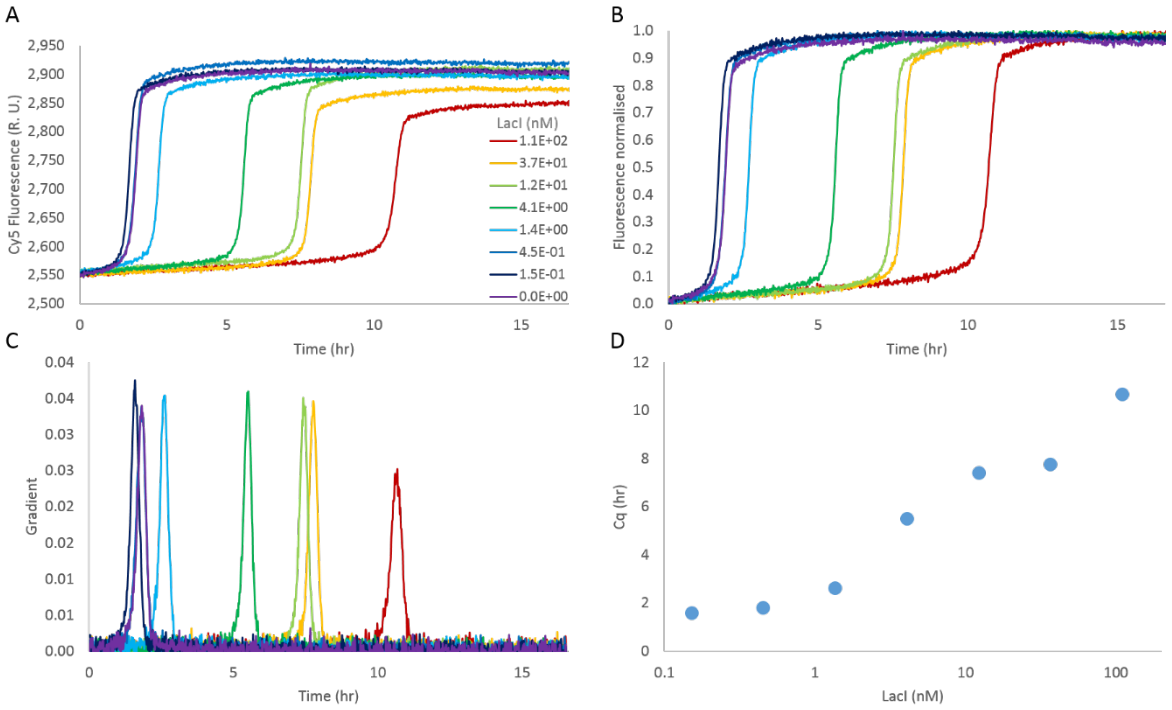

Supplementary Figure 5. Data processing workflow for Figure 1F. (**A**) Raw fluorescence reading for reaction assemblies containing varying concentrations of LacI dimer (in nM). (**B**) Same data as in **A**, but normalised between 1 and 0. (**C**) Derivatives of the curves in **B**. (**D**) Highest derivative time points for each LacI concentration.

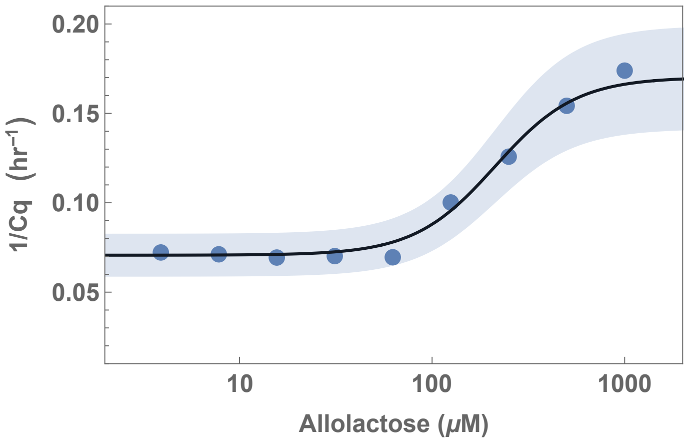

Supplementary Figure 6. Time response to a range of allolactose of psT$_{LacI}$-based sensor. Conditions as in Figure 1G.

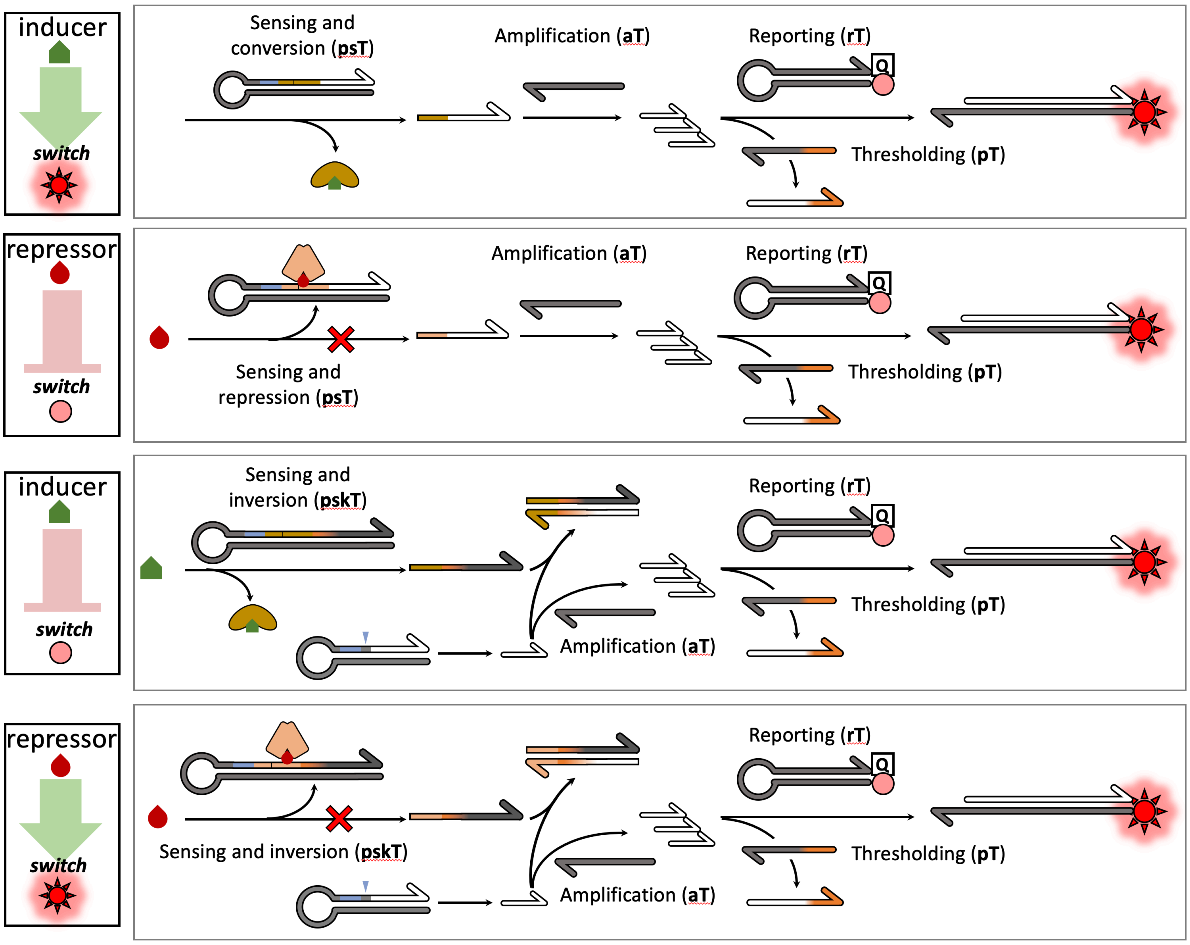

Supplementary Figure 7. Extended version of Figure 2. Mechanisms for signal propagation or inversion, depending on the logic of the allosteric transcription factor.

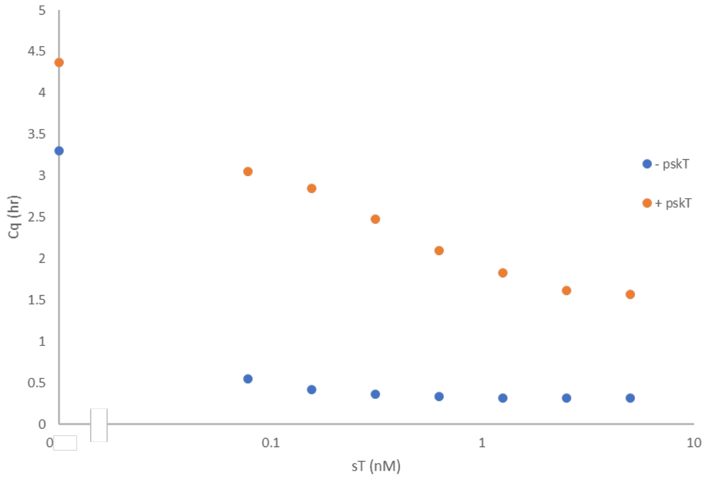

Supplementary Figure 8. Optimizing the sT concentration. We illustrate here the procedure used to determine the concentration of sT that we used throughout the work. For this we carried out a serial dilution of sT, with samples containing or not 5 nM of $pskT_{LacI}$. We were expecting to see a point where sT concentration would be sufficient to activate the switch, yet would be repressible. We found that a concentration of 8 pM – the minimal attempted – activated the switch with a small delay compared to higher concentrations, for which the triggering effect tended to saturate. In addition, lower concentrations of the sT correlated to better repression by pskT. Therefore, we have chosen the lowest concentration tested. We did not go lower as in potential future emulsion work lower concentrations can be problematic.

Supplementary Note 3. On effect of TrpR concentration on apparent K_d of pskT-based sensor.

In this work, we did not investigate systematically the impact of TF concentration on the circuit response. However, we expect it to affect the apparent K_d. In **Supp. Fig. 9**, we compare the response to L-trp for two TrpR concentrations, in the case of the inverted design with pskT (positive response). The 2-fold dilution notably affects apparent K_d which increases, as expected, but also the response becomes sharper. A further dilution to 42 nM resulted in loss of discrimination of the circuit as pskT repression became insufficient to overcome inhibition for all L-trp concentrations (not shown). This tuning opens up the possibility to adjust the sensing range and dynamics, depending on the application.

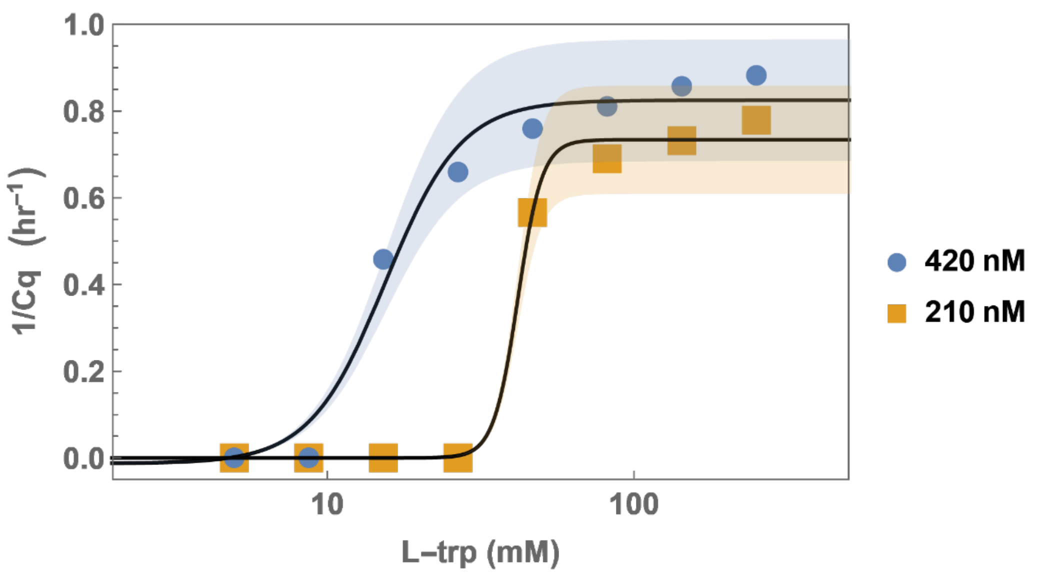

Supplementary Figure 9. Effect of TrpR concentration on apparent K_d of the L-trp pskT-based sensor. Final TrpR concentration of 420 nM (circles), 210 nM (squares). Conditions as in Figure 2F, apart from TrpR concentration.

Supplementary Note 4. Alternative sensing function shapes to complement Figure 3.

By adjusting the experimental conditions, it is possible to obtain various shapes for the response of a system to its two inputs. For example, compared to the system shown in **Figure 3D**, reproduced here as **Supp. Fig. 10A**, increasing pT concentration from 1nM to 4nM, setting a higher threshold, as well as decreasing psT_{TrpR} from 62.5 pM to 12.5pM, sets a much sharper cutoff in both dimensions (**Supp. Fig. 10B**).

In **Supp. Fig 10C, D** conditions were similar to **Supp. Fig 10B**, and only sensing module concentration was varied: psT_{TrpR}=125 pM, $pskT_{LacI}$=7.5 nM and psT_{TrpR}=62.5 pM, $pskT_{LacI}$=10 nM respectively. The resulting slow system in **D** becomes almost insensitive to IPTG input, whatever the L-trp concentration. the system in **C** applies a larger weight to L-trp, and a smaller to IPTG, compared to the original design.

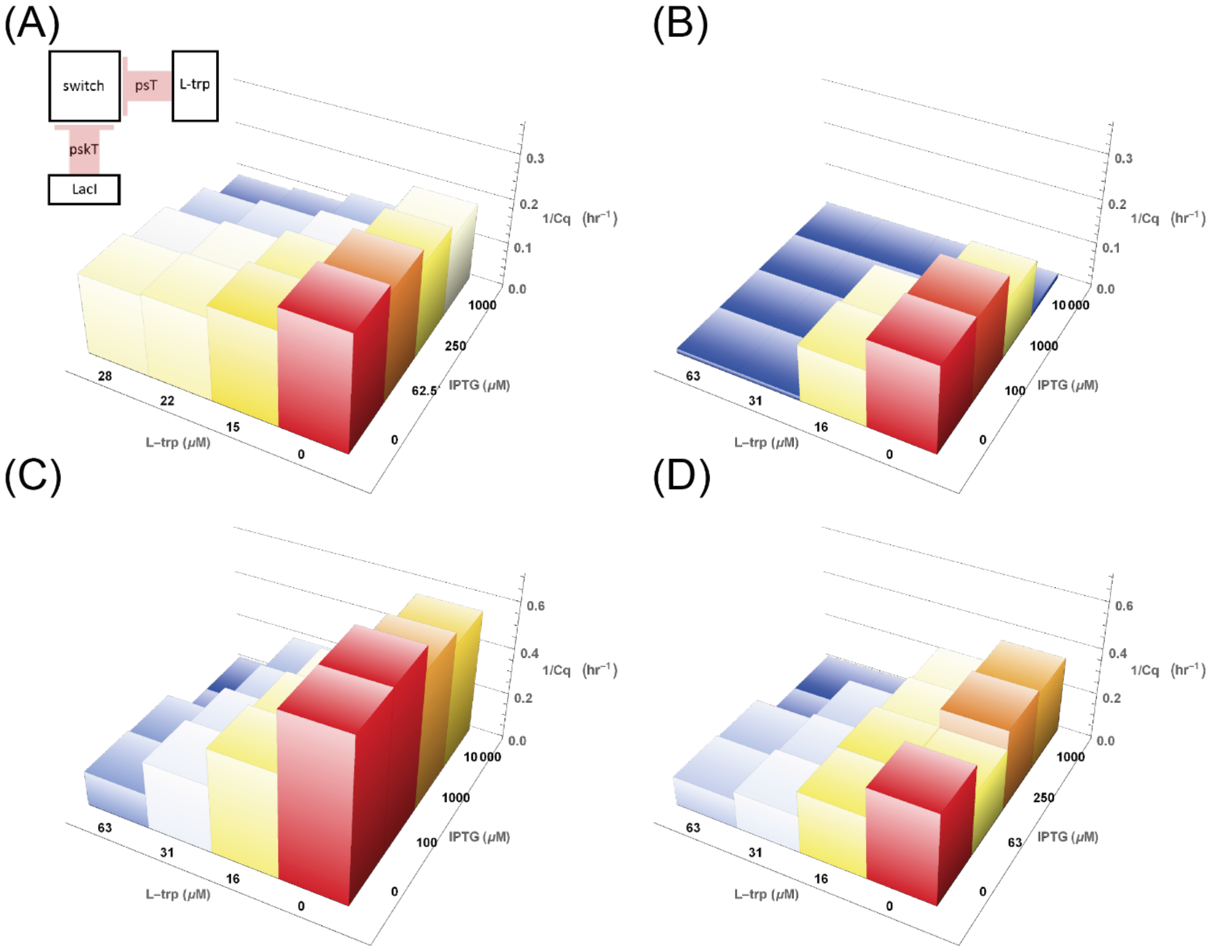

Supplementary Figure 10. Alternative plots for Figure 3D with altered input balance. (**A**) Original plot: pT=1 nM, psT_{TrpR}=0.063 nM, $pskT_{LacI}$=40 nM. (**B**) As in **A** bar pT=4nM, psT_{TrpR}=12.5 pM, $pskT_{LacI}$=10 nM. (**C**) As in **B** bar psT_{TrpR}=125 pM, $pskT_{LacI}$=7.5 nM (**D**) As in **B** bar psT_{TrpR}=62.5 pM, $pskT_{LacI}$=10 nM.

pskTs tend to provide sharper transitions implementing binary classification (**Supp. Fig. 11A**). The system is very sensitive as doubling sT concentration (from 8 to 16 pM) has a large impact on the classification limits (**Supp. Fig. 11B**). To obtain the smooth log-linear summing, we used 8 pM sT but rebalanced $pskT_{TrpR}$, $pskT_{LacI}$, from 5 nM of each to 7.5 nM of $pskT_{LacI}$ and 2.5 nM of $pskT_{TrpR.}$ This corrects the bias towards stronger TrpR-based element and brings more samples into the window of observation as the total amount of pskT leads to less repression (taking into account that LacI-based elements are less efficient) (**Supp. Fig. 11C**). Finally, we made a fine adjustment to $pskT_{TrpR}$=1.5nM, $pskT_{LacI}$=7.5nM, to further rebalance and speed up the switch to produce the final plot reported.

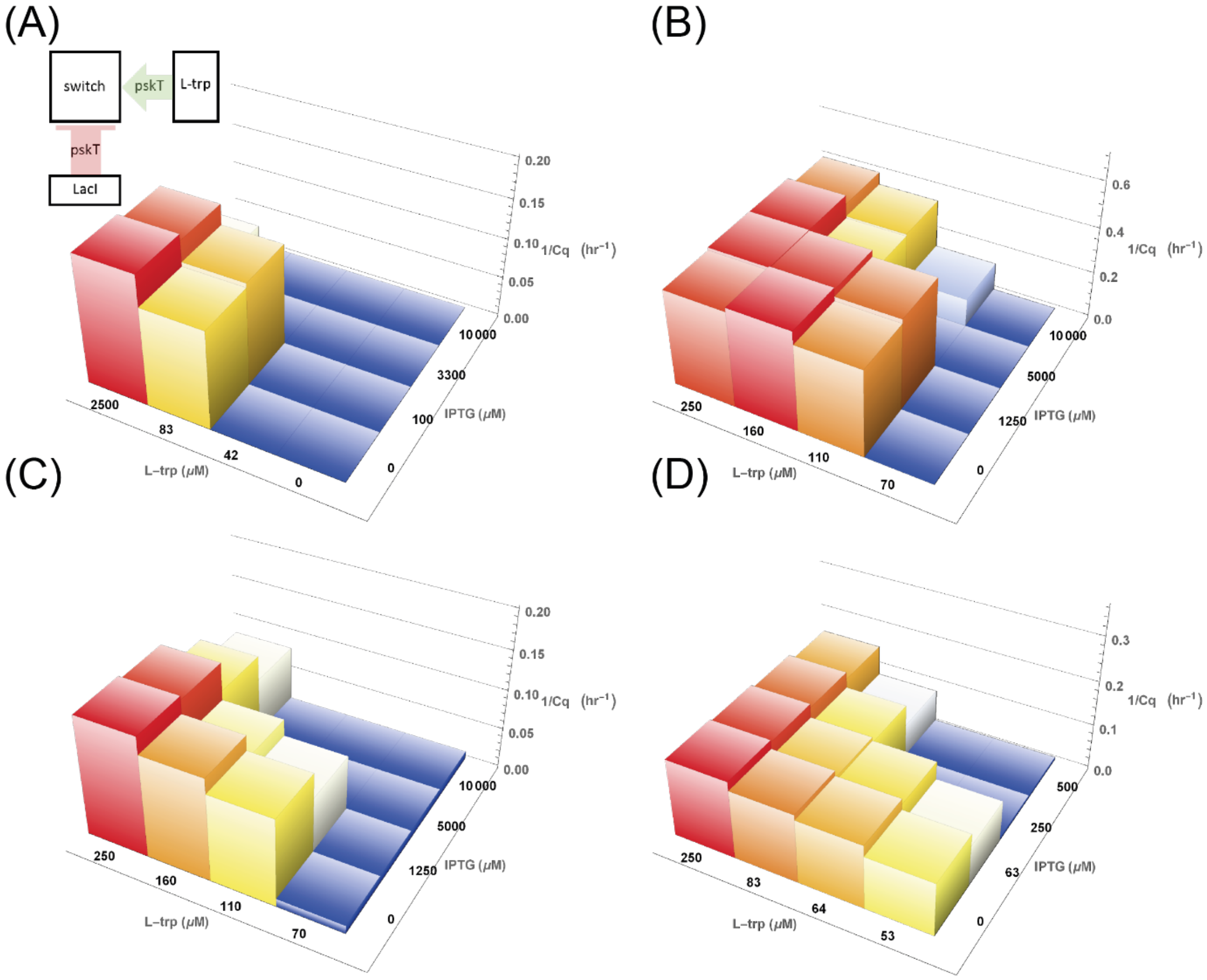

Supplementary Figure 11. Alternative shapes for the positive/negative circuit with altered input balance. (**A**) As in **D** bar $pskT_{TrpR}$=5 nM, $pskT_{LacI}$=5 nM. (**B**) As in **A** bar sT=16 pM. (**C**) As in **A** bar $pskT_{TrpR}$=2.5 nM, $pskT_{LacI}$=7.5 nM. (**D**) Original plot: pT=4 nM, $pskT_{TrpR}$=1.5 nM, $pskT_{LacI}$ 7.5 nM, sT=8 pM.

The positive/positive design shown in **Figure 3A** mixes psT and pskT. **Supp. Fig. 12A** shows a system starting too fast for input discrimination to occur. Decreasing the amount of psT_{LacI} ten-fold from 2.5 nM slows down the system, but makes it little sensitive to IPTG (**Supp. Fig. 12B**). Another option is to enter the dynamic range is to increase pT (from 4 nM to 7 nM in **Supp. Fig. 12C**). This provides a reasonable window of observation, which we narrowed down to arrive to the final plot reported (**Supp. Fig. 12D**).

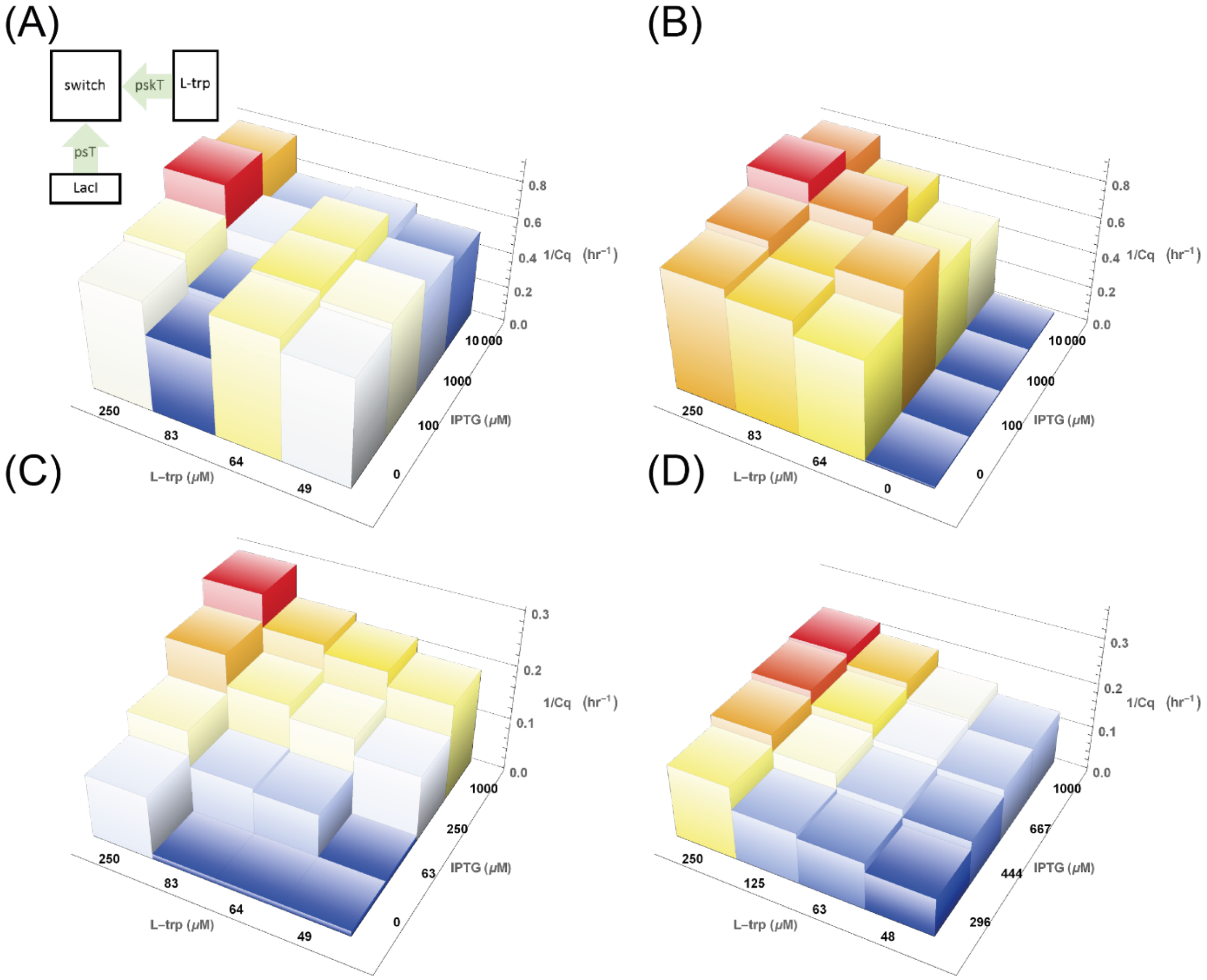

Supplementary Figure 12. Alternative plots for the positive/positive circuit with altered input balance. (**A**) As in **D** bar, pT=4nM $pskT_{TrpR}$=10 nM, psT_{LacI}=2.5 nM. (**B**) As in **A** bar psT_{LacI}=0.25 nM. (**C**) As in **A** bar pT=7nM. (**D**) Original plot: pT=7nM, $pskT_{TrpR}$ 10=nM, psT_{LacI}=10 nM.

Supplementary Note 5. Enzyme activity detection and gene amplification response.

In the section we wish to have the output of the PEN-DNA circuit, instead of producing a fluorescent signal, to enable the amplification of a full-length protein-encoding gene. The goal is to obtain an *enzymatic activity-to-genetic amplification* circuit that can be used to build autonomous selection networks for micro-compartmentalized directed evolution protocols. We thus replace (or complement) the reporter template by two primer-producing templates (ppT), which will use the signal B11 as input, and will produce two primers targeting a given gene. After isothermal incubation, primers are then produced according to the timing of ON-switching of the PEN circuit (with earlier switching leading to more primer accumulation), and can be used during a subsequent PCR. The primer-producing templates are designed as follows: 3' to 5', it contains the B11 binding site, a Nt.BstNBI recognition/nicking site with a 4 base pair spacer, and then the primer encoding sequence (**Supp. Fig. 10A**).

Following switch activation, B11 signal binds on ppTs and is extended. Cycle of nicking, product dissociation and polymerase extention will ensue, like previously described for psT. The result is a linear production and accumulation of primers, conditional to switch activation (**Supp. Fig. 10B**).

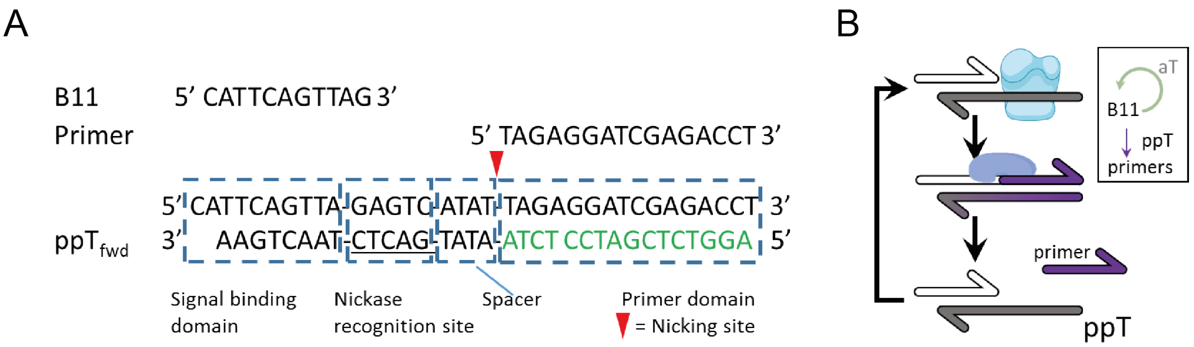

Supplementary Figure 13. Primer producing template (ppT) design. (**A**) On the bottom: single-stranded ppT_{fwd} is shown with B11 signal bond and extended. Going left to right it contains a signal binding domain, nickase recognition site, a spacer and primer-coding domain. (**B**) Schematic summary of primer production. Bound signal is extended, nicked and primer is released in cycles.

We tested a pair of ppTs (coding for a primer pair) to couple TrpB activity to gene amplification by PCR. After optimizing the conditions to enable enzymatic activity, isothermal circuit, and PCR in a single buffer, the experiment was conducted one pot, with ppTs at 10 nM and the target gene at 62 pM (see below for detailed reaction conditions). To be able to monitor the reaction in real time, we used both rT and ppT downstream of the circuit. We incubated the MP with a range of EcB concentrations at 37 ^{o}C. After full switch activation was observed for the fastest sample, we waited an additional 40 minutes (to enable primer production and switched the machine to PCR-cycling program.

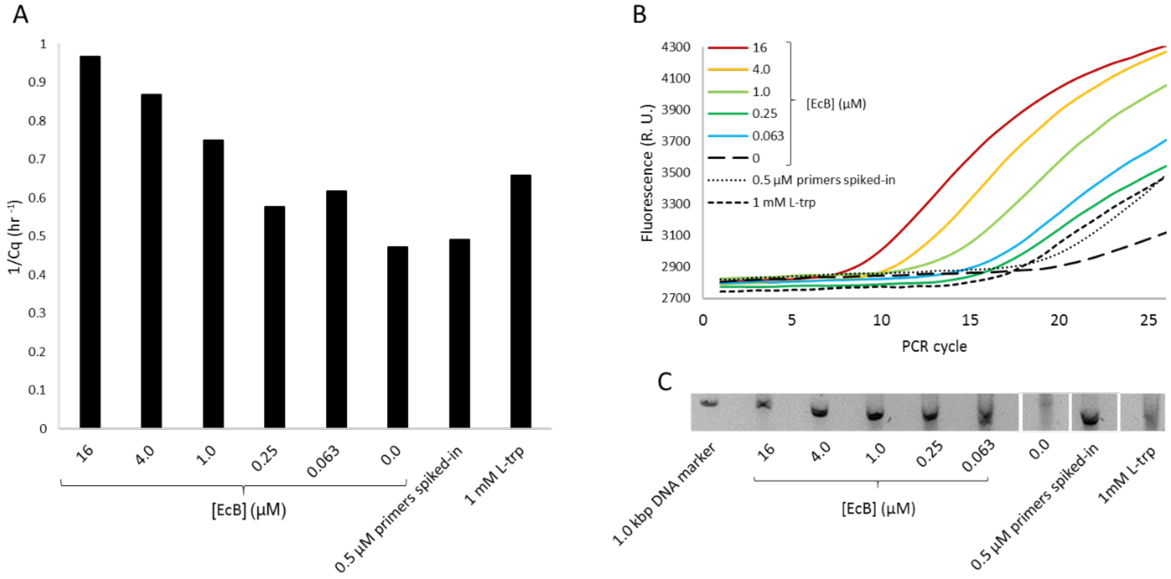

Supplementary Figure 14. EcB activity detection and gene amplification response. Samples consist of various concentrations of EcB, control with 0.5 µM primers spiked-in before PCR and 1 mM of L-trp added instead of the enzyme. (**A**) 1/Cq extracted during molecular program run. (**B**) real-time PCR amplification trace of PCR launched 40 min after first complete start in **A**. (**C**) Section of agarose gel of the PCR products retrieved from reactions shown in **A-B** along with a 1.0 kbp DNA ladder. "0.0" and "0.5 µM primers spiked-in" lanes were switched around horizontally through graphical editing for ease on interpretation. All samples were run on the same gel.

The PCR reaction was followed in real time thanks to an intercalating dye. We indeed observed a correlation between switching time and real-time PCR amplification rate (**Supp. Fig. 11A-B**). We also checked by agarose gel electrophoresis that the PCR reaction produced the expected amplification product **(Supp. Fig.11C)**. Overall, this proves that we chemically link a catalytic activity, orthogonal to DNA, to gene amplification.

Method

In this section, a modified assembly was used. The buffer contained 20 mM Tris-HCl pH8.9, 5 mM $(NH_4)_2SO_4$, 20 mM KCl, 7 mM $MgSO_4$, 5 mM NaCl, 2 µM Netropsin (Sigma-Aldrich), 200 µg ml^{-1} BSA (New England Biolabs, NEB), 1% Triton X-100 (Sigma-Aldrich), 0.4X EvaGreen dye (Biotium) and dNTPs (200 µM each). As before, the mixture also contained Bst DNA polymerase, large fragment, the nicking endonucleases Nb.BsmI and Nt.BstNBI (all from NEB) respectively used at 20, 200 and 50 U ml^{-1} (respectively 0.25, 2 and 0.5% final dilutions of the commercial stock solutions). The thermophilic 5' -> 3' exonuclease ttRecJ was purified in the laboratory, stored in Diluent A (NEB) + 0.1% Triton X-100 at a concentration of 1.53 µM and used at 22.95nM. In addition, Vent (exo-) from NEB was used at 20 U ml^{-1} or 1% of the commercial stock, 50 nM of TrpR dimer, 5 mM of L-serine, 80 µM PLP, 2.5mM indole, 62pM sfGFP-template plasmid (p070 – see **Table of vectors and genes**). As well as 90 nM aT, 50 nM rT, 15 nM pT, 70pM sT, 10 nM pskT$_{TrpR}$, 10nM ppT$_{fwd}$, 10nM ppT$_{rev}$. Also, we extracted Cq values based on passing a fluorescence threshold of 40% of maximum relative fluorescence increase as the switching did not fully occur for all samples. PCR protocol consisted of 95°C incubation for 2.5 min, followed by 25 cycles of 95°C-15s, 61°C-30s, 72°C-60s, and final 72°C extension for 5min.

Novel oligos used in molecular programs of this paper.

Part name	Sequence
B11	CATTCAGTTAG
aT	C*T*A*ACTGAATG-CTAACTGAA
pT	A*A*-C*TAACTGAATG
rT	Atto663-C*A*T*CGATATACTAACTGAATGCGATG-BHQ2
psT_{TrpR}	TG-CTAACTGAA-CGAACTAGTTAACTAGTAC-GACTC-TGCAAT-TTTTT-ATTGCAGAGTCGTACTAGTTAACTAGTTCGTTCA
psT_{LacI}	TG-CTAACTGAA-CAATTGTGAGCGGATAACAATT-GACTC-TGCAAT-TTTTT-ATTGCAGAGTCAATTGTTATCCGCTCACAATTGTTCA
$pskT_{LacI}$	CATTCAGTTAGTT-CAATTGTGAGCGGATAACAATT-GACTC-TGCAAT TTTTT ATTGCAGAGTCAATTGTTATCCGCTCACAATTGAACTAACTGAATG
$pskT_{TrpR}$	CATTCAGTTAGTT-CGAACTAGTTAACTAGTAC-GACTC-TGCAAT-TTTTT-ATTGCAGAGTCGTACTAGTTAACTAGTTCGAACT
sT	TG-CTAACTGAA-C-GACTC-TGC-TTTTT-GCAGAGTCGTTCA
ppT_{fwd}	AGGTCTCGATCCTCTAATATGACTCTAACTGAA
ppT_{rev}	GCACCGTATCATCTTCATATGACTCTAACTGAA

Supplementary Table 2 Novel oligos used in molecular programs of this paper. Stars represent phosphorothioate modification. Fluorophore and quencher modification positions are shown for rT. Dashes are shown for ease of interpretation.

Supplementary Note 6. Cloning materials.

NEB 5-alpha annotated genome (GenBank: CP017100.1) was used for *E.coli*-sourced parts. *PfB* sequence was inferred from UniProt (Q8U093 (TRPB1_PYRFU)), adjusted by IDT for codon optimisation and synthesis constraints and ordered as gBlock. *TrpR*, *LacI* and *TrpB* coding sequences were cloned into pIVEX vector containing Thrombin cleavage site and His-tag on the C-terminus via Gibson assembly using primers shown below.

Table of Vectors and genes

Name	Sequence
PfB ordered as gBlock	ATGTGGTTCGGTGAGTTTGGTGGGCAGTATGTCCCGGAAACGCTTATTGAACCG<br>CTGAAGGAGCTGGAAAAGGCATATAAGCGGTTCAAAGATGATGAAGAGTTCAA<br>CCGCCAACTCAACTATTACTTAAAAACATGGGCTGGTCGCCCAACTCCGCTGTATT<br>ATGCTAAGCGTCTTACGGAAAAGATTGGGGGTGCAAAAATCTACCTGAAGCGTG<br>AAGACTTGGTGCACGGGGGCGCTCATAAAACAAATAACGCGATTGGCCAGGCTT<br>TGCTCGCAAAATTTATGGGTAAGACCCGCTTAATTGCTGAGACAGGCGCAGGTC<br>AACATGGCGTAGCCACAGCGATGGCTGGCGCGTTATTAGGCATGAAGGTAGATA<br>TCTATATGGGTGCTGAAGACGTAGAGCGCCAGAAGATGAACGTTTTTCGCATGA<br>AGTTGTTGGGGGCGAATGTAATTCCAGTCAACAGTGGCTCACGCACTTTGAAAG<br>ATGCCATTAATGAAGCATTACGGGACTGGGTAGCCACCTTCGAGTACACGCACTA<br>CCTGATTGGCTCTGTCGTCGGCCCTCATCCGTATCCTACTATTGTGCGCGACTTCC<br>AGAGCGTCATTGGCCGTGAAGCCAAAGCACAAATCCTGGAGGCAGAGGGCCAG<br>TTGCCTGACGTCATTGTCGCCTGTGTTGGGGGCGGGTCAAACGCTATGGGGATC<br>TTTTACCCGTTTGTTAATGATAAAAAAGTCAAACTCGTTGGCGTGGAAGCTGGCG<br>GCAAGGGGCTTGAGAGTGGCAAACATTCGGCAAGCTTAAATGCTGGGCAGGTT<br>GGGGTATTTCATGGCATGTTATCTTACTTCTTACAGGACGAGGAGGGGCAAATC<br>AAGCCAACACACTCGATTGCGCCGGGGCTGGATTACCCTGGTGTCGGGCCGGAA<br>CACGCTTACCTGAAAAAAATCCAACGTGCGGAGTACGTGACTGTCACAGATGAG<br>GAAGCGCTCAAAGCCTTCCACGAATTATCACGTACCGAAGGGATCATCCCGGCTC<br>TGGAGTCAGCACATGCCGTCGCGTACGCTATGAAGTTGGCGAAAGAGATGTCAC<br>GTGATGAAATCATTATTGTAAACTTGAGTGGTCGGGGCGATAAAGACCTCGACA<br>TCGTCCTGAAAGTCAGCGGTAATGTG
His-pIVEX vector used in this work	CTGGTGCCGCGTGGCAGCTTACATCCCGGGGGGGGTTCTCATCATCATCATCATC<br>ATTAATAAAAGGGCGAATTCCAGCACACTGGCGGCCGTTACTAGTGGATCCGGC<br>TGCTAACAAAGCCCGAAAGGAAGCTGAGTTGGCTGCTGCCACCGCTGAGCAATA<br>ACTAGCATAACCCCTTGGGGCCTCTAAACGGGTCTTGAGGGGTTTTTTGCTGAAA<br>GGAGGAACTATATCCGGATATCCACAGGACGGGTGTGGTCGCCATGATCGCGTA<br>GTCGATAGTGGCTCCAAGTAGCGAAGCGAGCAGGACTGGGCGGCGGCCAAAGC<br>GGTCGGACAGTGCTCCGAGAACGGGTGCGCATAGAAATTGCATCAACGCATATA<br>GCGCTAGCAGCACGCCATAGTGACTGGCGATGCTGTCGGAATGGACGATATCCC<br>GCAAGAGGCCCGGCAGTACCGGCATAACCAAGCCTATGCCTACAGCATCCAGGG<br>TGACGGTGCCGAGGATGACGATGAGCGCATTGTTAGATTTCATACACGGTGCCT<br>GACTGCGTTAGCAATTTAACTGTGATAAACTACCGCATTAAAGCTTATCGATGAT<br>AAGCTGTCAAACATGAGAATTCGTAATCATGTCATAGCTGTTTCCTGTGTGAAAT<br>TGTTATCCGCTCACAATTCCACACAACATACGAGCCGGAAGCATAAAGTGTAAAG<br>CCTGGGGTGCCTAATGAGTGAGCTAACTCACATTAATTGCGTTGCGCTCACTGCC<br>CGCTTTCCAGTCGGGAAACCTGTCGTGCCAGCTGCATTAATGAATCGGCCAACGC<br>GCGGGGAGAGGCGGTTTGCGTATTGGGCGCTCTTCCGCTTCCTCGCTCACTGACT<br>CGCTGCGCTCGGTCGTTCGGCTGCGGCGAGCGGTATCAGCTCACTCAAAGGCGG<br>TAATACGGTTATCCACAGAATCAGGGGATAACGCAGGAAAGAACATGTGAGCAA<br>AAGGCCAGCAAAAGGCCAGGAACCGTAAAAAGGCCGCGTTGCTGGCGTTTTTCC<br>ATAGGCTCCGCCCCCCTGACGAGCATCACAAAAATCGACGCTCAAGTCAGAGGT<br>GGCGAAACCCGACAGGACTATAAAGATACCAGGCGTTTCCCCCTGGAAGCTCCC<br>TCGTGCGCTCTCCTGTTCCGACCCTGCCGCTTACCGGATACCTGTCCGCCTTTCTC<br>CCTTCGGGAAGCGTGGCGCTTTCTCATAGCTCACGCTGTAGGTATCTCAGTTCGG

	TGTAGGTCGTTCGCTCCAAGCTGGGCTGTGTGCACGAACCCCCCGTTCAGCCCGA<br>CCGCTGCGCCTTATCCGGTAACTATCGTCTTGAGTCCAACCCGGTAAGACACGAC<br>TTATCGCCACTGGCAGCAGCCACTGGTAACAGGATTAGCAGAGCGAGGTATGTA<br>GGCGGTGCTACAGAGTTCTTGAAGTGGTGGCCTAACTACGGCTACACTAGAAGG<br>ACAGTATTTGGTATCTGCGCTCTGCTGAAGCCAGTTACCTTCGGAAAAAGAGTTG<br>GTAGCTCTTGATCCGGCAAACAAACCACCGCTGGTAGCGGTGGTTTTTTTGTTTG<br>CAAGCAGCAGATTACGCGCAGAAAAAAAGGATCTCAAGAAGATCCTTTGATCTT<br>TTCTACGGGGTCTGACGCTCAGTGGAACGAAAACTCACGTTAAGGGATTTTGGT<br>CATGAGATTATCAAAAAGGATCTTCACCTAGATCCTTTTAAATTAAAAATGAAGTT<br>TTAAATCAATCTAAAGTATATATGAGTAAACTTGGTCTGACAGTTACCAATGCTTA<br>ATCAGTGAGGCACCTATCTCAGCGATCTGTCTATTTCGTTCATCCATAGTTGCCTG<br>ACTCCCCGTCGTGTAGATAACTACGATACGGGAGGGCTTACCATCTGGCCCCAGT<br>GCTGCAATGATACCGCGAGACCCACGCTCACCGGCTCCAGATTTATCAGCAATAA<br>ACCAGCCAGCCGGAAGGGCCGAGCGCAGAAGTGGTCCTGCAACTTTATCCGCCT<br>CCATCCAGTCTATTAATTGTTGCCGGGAAGCTAGAGTAAGTAGTTCGCCAGTTAA<br>TAGTTTGCGCAACGTTGTTGCCATTGCTACAGGCATCGTGGTGTCACGCTCGTCG<br>TTTGGTATGGCTTCATTCAGCTCCGGTTCCCAACGATCAAGGCGAGTTACATGAT<br>CCCCCATGTTGTGCAAAAAAGCGGTTAGCTCCTTCGGTCCTCCGATCGTTGTCAG<br>AAGTAAGTTGGCCGCAGTGTTATCACTCATGGTTATGGCAGCACTGCATAATTCT<br>CTTACTGTCATGCCATCCGTAAGATGCTTTTCTGTGACTGGTGAGTACTCAACCAA<br>GTCATTCTGAGAATAGTGTATGCGGCGACCGAGTTGCTCTTGCCCGGCGTCAATA<br>CGGGATAATACCGCGCCACATAGCAGAACTTTAAAAGTGCTCATCATTGGAAAA<br>CGTTCTTCGGGGCGAAAACTCTCAAGGATCTTACCGCTGTTGAGATCCAGTTCGA<br>TGTAACCCACTCGTGCACCCAACTGATCTTCAGCATCTTTTACTTTCACCAGCGTTT<br>CTGGGTGAGCAAAAACAGGAAGGCAAAATGCCGCAAAAAAGGGAATAAGGGC<br>GACACGGAAATGTTGAATACTCATACTCTTCCTTTTTCAATATTATTGAAGCATTT<br>ATCAGGGTTATTGTCTCATGAGCGGATACATATTTGAATGTATTTAGAAAAATAA<br>ACAAATAGGGGTTCCGCGCACATTTCCCCGAAAAGTGCCACCTGACGTCTAAGA<br>AACCATTATTATCATGACATTAACCTATAAAAATAGGCGTATCACGAGGCCCTTTC<br>GTCTCGCGCGTTTCGGTGATGACGGTGAAAACCTCTGACACATGCAGCTCCCGG<br>AGACGGTCACAGCTTGTCTGTAAGCGGATGCCGGGAGCAGACAAGCCCGTCAG<br>GGCGCGTCAGCGGGTGTTGGCGGGTGTCGGGGCTGGCTTAACTATGCGGCATC<br>AGAGCAGATTGTACTGAGAGTGCACCATATATGCGGTGTGAAATACCGCACAGA<br>TGCGTAAGGAGAAAATACCGCATCAGGCGCCATTCGCCATTCAGGCTGCGCAAC<br>TGTTGGGAAGGGCGATCGGTGCGGGCCTCTTCGCTATTACGCCAGCTGGCGAAA<br>GGGGGATGTGCTGCAAGGCGATTAAGTTGGGTAACGCCAGGGTTTTCCCAGTCA<br>CGACGTTGTAAAACGACGGCCAGTGCCAAGCTTGCATGCAAGGAGATGGCGCCC<br>AACAGTCCCCCGGCCACGGGGCCTGCCACCATACCCACGCCGAAACAAGCGCTC<br>ATGAGCCCGAAGTGGCGAGCCCGATCTTCCCCATCGGTGATGTCGGCGATATAG<br>GCGCCAGCAACCGCACCTGTGGCGCCGGTGATGCCGGCCACGATGCGTCCGGC<br>GTAGAGGATCGAGATCTCGATCCCGCGAAATTAATACGACTCACTATAGGGAGA<br>CCACAACGGTTTCCCTCTAGAAATAATTTTGTTTAACTTTAAGAAGGAGATATACC

p070 plasmid provided by Rémi Sieskind, containing sfGFP	CTAGCATAACCCCTTGGGGCCTCTAAACGGGTCTTGAGGGGTTTTTTGCTGAAAG<br>GAGGAACTATATCCGGATATCCACAGGACGGGTGTGGTCGCCATGATCGCGTAG<br>TCGATAGTGGCTCCAAGTAGCGAAGCGAGCAGGACTGGGCGGCGGCCAAAGCG<br>GTCGGACAGTGCTCCGAGAACGGGTGCGCATAGAAATTGCATCAACGCATATAG<br>CGCTAGCAGCACGCCATAGTGACTGGCGATGCTGTCGGAATGGACGATATCCCG<br>CAAGAGGCCCGGCAGTACCGGCATAACCAAGCCTATGCCTACAGCATCCAGGGT<br>GACGGTGCCGAGGATGACGATGAGCGCATTGTTAGATTTCATACACGGTGCCTG<br>ACTGCGTTAGCAATTTAACTGTGATAAACTACCGCATTAAAGCTTATCGATGATA<br>AGCTGTCAAACATGAGAATTCGTAATCATGTCATAGCTGTTTCCTGTGTGAAATT<br>GTTATCCGCTCACAATTCCACACAACATACGAGCCGGAAGCATAAAGTGTAAAGC<br>CTGGGGTGCCTAATGAGTGAGCTAACTCACATTAATTGCGTTGCGCTCACTGCCC<br>GCTTTCCAGTCGGGAAACCTGTCGTGCCAGCTGCATTAATGAATCGGCCAACGC<br>GCGGGGAGAGGCGGTTTGCGTATTGGGCGCTCTTCCGCTTCCTCGCTCACTGACT<br>CGCTGCGCTCGGTCGTTCGGCTGCGGCGAGCGGTATCAGCTCACTCAAAGGCGG<br>TAATACGGTTATCCACAGAATCAGGGGATAACGCAGGAAAGAACATGTGAGCAA<br>AAGGCCAGCAAAAGGCCAGGAACCGTAAAAAGGCCGCGTTGCTGGCGTTTTTCC<br>ATAGGCTCCGCCCCCCTGACGAGCATCACAAAAATCGACGCTCAAGTCAGAGGT<br>GGCGAAACCCGACAGGACTATAAAGATACCAGGCGTTTCCCCCTGGAAGCTCCC<br>TCGTGCGCTCTCCTGTTCCGACCCTGCCGCTTACCGGATACCTGTCCGCCTTTCTC<br>CCTTCGGGAAGCGTGGCGCTTTCTCATAGCTCACGCTGTAGGTATCTCAGTTCGG<br>TGTAGGTCGTTCGCTCCAAGCTGGGCTGTGTGCACGAACCCCCCGTTCAGCCCGA<br>CCGCTGCGCCTTATCCGGTAACTATCGTCTTGAGTCCAACCCGGTAAGACACGAC<br>TTATCGCCACTGGCAGCAGCCACTGGTAACAGGATTAGCAGAGCGAGGTATGTA<br>GGCGGTGCTACAGAGTTCTTGAAGTGGTGGCCTAACTACGGCTACACTAGAAGG<br>ACAGTATTTGGTATCTGCGCTCTGCTGAAGCCAGTTACCTTCGGAAAAAGAGTTG<br>GTAGCTCTTGATCCGGCAAACAAACCACCGCTGGTAGCGGTGGTTTTTTTGTTTG<br>CAAGCAGCAGATTACGCGCAGAAAAAAAGGATCTCAAGAAGATCCTTTGATCTT<br>TTCTACGGGGTCTGACGCTCAGTGGAACGAAAACTCACGTTAAGGGATTTTGGT<br>CATGAGATTATCAAAAAGGATCTTCACCTAGATCCTTTTAAATTAAAAATGAAGTT<br>TTAAATCAATCTAAAGTATATATGAGTAAACTTGGTCTGACAGTTACCAATGCTTA<br>ATCAGTGAGGCACCTATCTCAGCGATCTGTCTATTTCGTTCATCCATAGTTGCCTG<br>ACTCCCCGTCGTGTAGATAACTACGATACGGGAGGGCTTACCATCTGGCCCCAGT<br>GCTGCAATGATACCGCGAGACCCACGCTCACCGGCTCCAGATTTATCAGCAATAA<br>ACCAGCCAGCCGGAAGGGCCGAGCGCAGAAGTGGTCCTGCAACTTTATCCGCCT<br>CCATCCAGTCTATTAATTGTTGCCGGGAAGCTAGAGTAAGTAGTTCGCCAGTTAA<br>TAGTTTGCGCAACGTTGTTGCCATTGCTACAGGCATCGTGGTGTCACGCTCGTCG<br>TTTGGTATGGCTTCATTCAGCTCCGGTTCCCAACGATCAAGGCGAGTTACATGAT<br>CCCCCATGTTGTGCAAAAAAGCGGTTAGCTCCTTCGGTCCTCCGATCGTTGTCAG<br>AAGTAAGTTGGCCGCAGTGTTATCACTCATGGTTATGGCAGCACTGCATAATTCT<br>CTTACTGTCATGCCATCCGTAAGATGCTTTTCTGTGACTGGTGAGTACTCAACCAA<br>GTCATTCTGAGAATAGTGTATGCGGCGACCGAGTTGCTCTTGCCCGGCGTCAATA<br>CGGGATAATACCGCGCCACATAGCAGAACTTTAAAAGTGCTCATCATTGGAAAA<br>CGTTCTTCGGGGCGAAAACTCTCAAGGATCTTACCGCTGTTGAGATCCAGTTCGA<br>TGTAACCCACTCGTGCACCCAACTGATCTTCAGCATCTTTTACTTTCACCAGCGTTT<br>CTGGGTGAGCAAAAACAGGAAGGCAAAATGCCGCAAAAAAGGGAATAAGGGC<br>GACACGGAAATGTTGAATACTCATACTCTTCCTTTTTCAATATTATTGAAGCATTT<br>ATCAGGGTTATTGTCTCATGAGCGGATACATATTTGAATGTATTTAGAAAAATAA

	ACAAATAGGGGTTCCGCGCACATTTCCCCGAAAAGTGCCACCTGACGTCTAAGA<br>AACCATTATTATCATGACATTAACCTATAAAAATAGGCGTATCACGAGGCCCTTTC<br>GTCTCGCGCGTTTCGGTGATGACGGTGAAAACCTCTGACACATGCAGCTCCCGG<br>AGACGGTCACAGCTTGTCTGTAAGCGGATGCCGGGAGCAGACAAGCCCGTCAG<br>GGCGCGTCAGCGGGTGTTGGCGGGTGTCGGGGCTGGCTTAACTATGCGGCATC<br>AGAGCAGATTGTACTGAGAGTGCACCATATATGCGGTGTGAAATACCGCACAGA<br>TGCGTAAGGAGAAAATACCGCATCAGGCGCCATTCGCCATTCAGGCTGCGCAAC<br>TGTTGGGAAGGGCGATCGGTGCGGGCCTCTTCGCTATTACGCCAGCTGGCGAAA<br>GGGGGATGTGCTGCAAGGCGATTAAGTTGGGTAACGCCAGGGTTTTCCCAGTCA<br>CGACGTTGTAAAACGACGGCCAGTGCCAAGCTTGCATGCAAGGAGATGGCGCCC<br>AACAGTCCCCCGGCCACGGGGCCTGCCACCATACCCACGCCGAAACAAGCGCTC<br>ATGAGCCCGAAGTGGCGAGCCCGATCTTCCCCATCGGTGATGTCGGCGATATAG<br>GCGCCAGCAACCGCACCTGTGGCGCCGGTGATGCCGGCCACGATGCGTCCGGC<br>GTAGAGGATCGAGATCTCGATCCCGCGAAATTAATACGACTCACTATAGGGAGA<br>CCACAACGGTTTCCCTCTAGAAATAATTTTGTTTAACTTTAAGAAGGAGATATACC<br>ATGGAGCTTTTCACTGGCGTTGTTCCCATCCTGGTCGAGCTGGACGGCGACGTAA<br>ACGGCCACAAGTTCAGCGTGTCCGGCGAGGGCGAGGGCGATGCCACCTACGGC<br>AAGCTGACCCTGAAGTTCATCTGCACCACCGGCAAGCTGCCCGTGCCCTGGCCCA<br>CCCTCGTGACCACCCTGACCTACGGCGTGCAGTGCTTCAGCCGCTACCCCGACCA<br>CATGAAGCAGCACGACTTCTTCAAGTCCGCCATGCCCGAAGGCTACGTCCAGGA<br>GCGCACCATCTTCTTCAAGGACGACGGCAACTACAAGACCCGCGCCGAGGTGAA<br>GTTCGAGGGCGACACCCTGGTGAACCGCATCGAGCTGAAGGGCATCGACTTCAA<br>GGAGGACGGCAACATCCTGGGGCACAAGCTGGAGTACAACTACAACAGCCACA<br>ACGTCTATATCATGGCCGACAAGCAGAAGAACGGCATCAAGGTGAACTTCAAGA<br>TCCGCCACAACATCGAGGACGGCAGCGTGCAGCTCGCCGACCACTACCAGCAGA<br>ACACCCCCATCGGCGACGGCCCCGTGCTGCTGCCCGACAACCACTACCTGAGCAC<br>CCAGTCCGCCCTGAGCAAAGACCCCAACGAGAAGCGCGATCACATGGTCCTGCT<br>GGAGTTCGTGACCGCCGCCGGGATCGTACAGACCGGTGAATTCACCATGGACAA<br>AGACTGCGAAATGAAGCGCACCACCCTGGATAGCCCTCTGGGCAAGCTGGAACT<br>GTCTGGGTGCGAACAGGGCCTGCACCGTATCATCTTCCTGGGCAAAGGAACATC<br>TGCCGCCGACGCCGTGGAAGTGCCTGCCCCAGCCGCCGTGCTGGGCGGACCAG<br>AGCCACTGATGCAGGCCACCGCCTGGCTCAACGCCTACTTTCACCAGCCTGAGGC<br>CATCGAGGAGTTCCCTGTGCCAGCCCTGCACCACCCAGTGTTCCAGCAGGAGAG<br>CTTTACCCGCCAGGTGCTGTGGAAACTGCTGAAAGTGGTGAAGTTCGGAGAGGT<br>CATCAGCTACAGCCACCTGGCCGCCCTGGCCGGCAATCCCGCCGCCACCGCCGCC<br>GTGAAAACCGCCCTGAGCGGAAATCCCGTGCCCATTCTGATCCCCTGCCACCGG<br>GTGGTGCAGGGCGACCTGGACGTGGGGGGCTACGAGGGCGGGCTCGCCGTGA<br>AAGAGTGGCTGCTGGCCCACGAGGGCCACAGACTGGGCAAGCCTGGGCTGGGT<br>CCTGCAGGCGGATCCGCGTTTAAACTCGAGGTTAATTAATCCCGGGGGGGGTTC<br>TCATCATCATCATCATCATTAATAAAAGGGCGAATTCCAGCACACTGGCGGCCGT<br>TACTAGTGGATCCGGCTGCTAACAAAGCCCGAAAGGAAGCTGAGTTGGCTGCTG<br>CCACCGCTGAGCAATAA

Table of primers

Purpose	Name	Sequence
Vector amplification (pIVEX)	Filippo rev_020	TGGTATATCTCCTTCTTAAAGTTAAACAAAATTATTTCTAGAGGG
	iR280	CTGGTGCCGCGTGGCAGCTTACATCCCGGGGGGGTTCT
Vector integration	PfB2 REV	AAGCTGCCACGCGGCACCAGCACATTACCGCTGACTTTCA
	PfB2 FWD	CTTTAAGAAGGAGATATACCATGTGGTTCGGTGAGTTTGG
Point mutation introduction	T292S_R	TTGCCCCTCCTCGTCCTG
	T292S_F	ATCAAGCCAAGCCACTCGATTGCGCCGG
Vector integration	EcB REV2 non-stop	AAGCTGCCACGCGGCACCAGGATTTCCCCTCGTGCTT
	EcB FWD	CTTTAAGAAGGAGATATACCATGACAACATTACTTAACCCCT
Point mutation introduction	M149T_F	GTTTTTCGTACCCGCTTAATGGGTGCGGAAG
	M149T_R	GTTAGGCGACTGGCGTTC
	N171D_F	AGATGCCTGTGATGAGGCGCTGC
	N171D_R	TTCAGCGTCGCGGAACCG
Vector integration	GTrp FWD	CTTTAAGAAGGAGATATACCATGGCCCAACAATCACCCTA
	GTrp REV	AAGCTGCCACGCGGCACCAGATCGCTTTTCAGCAACACCT
Vector integration	GLacI FWD	CTTTAAGAAGGAGATATACCATGGTGAATGTGAAACCAGTAAC
	GLacI REV	AAGCTGCCACGCGGCACCAGCTGCCCGCTTTCCAGTC
sfGFP amplification control	iR338	GAAGATGATACGGTGC
	iR337	TAGAGGATCGAGACCT
